# Structure from motion photogrammetry in ecology: Does the choice of software matter?

**DOI:** 10.1002/ece3.5443

**Published:** 2019-09-30

**Authors:** Joel Forsmoo, Karen Anderson, Christopher J. A. Macleod, Mark E. Wilkinson, Leon DeBell, Richard E. Brazier

**Affiliations:** ^1^ Environment and Sustainability Institute University of Exeter Penryn UK; ^2^ James Hutton Institute Aberdeen UK; ^3^ Geography University of Exeter Exeter UK

**Keywords:** drone, elevation model, photogrammetry, reproducibility, structure from motion and multi‐view stereo, sward height

## Abstract

Image‐based modeling, and more precisely, Structure from Motion (SfM) and Multi‐View Stereo (MVS), is emerging as a flexible, self‐service, remote sensing tool for generating fine‐grained digital surface models (DSMs) in the Earth sciences and ecology. However, drone‐based SfM + MVS applications have developed at a rapid pace over the past decade and there are now many software options available for data processing. Consequently, understanding of reproducibility issues caused by variations in software choice and their influence on data quality is relatively poorly understood. This understanding is crucial for the development of SfM + MVS if it is to fulfill a role as a new quantitative remote sensing tool to inform management frameworks and species conservation schemes. To address this knowledge gap, a lightweight multirotor drone carrying a Ricoh GR II consumer‐grade camera was used to capture replicate, centimeter‐resolution image datasets of a temperate, intensively managed grassland ecosystem. These data allowed the exploration of method reproducibility and the impact of SfM + MVS software choice on derived vegetation canopy height measurement accuracy. The quality of DSM height measurements derived from four different, yet widely used SfM‐MVS software—Photoscan, Pix4D, 3DFlow Zephyr, and MICMAC, was compared with in situ data captured on the same day as image capture. We used both traditional agronomic techniques for measuring sward height, and a high accuracy and precision differential GPS survey to generate independent measurements of the underlying ground surface elevation. Using the same replicate image dataset (*n* = 3) as input, we demonstrate that there are 1.7, 2.0, and 2.5 cm differences in RMSE (excluding one outlier) between the outputs from different SfM + MVS software using High, Medium, and Low quality settings, respectively. Furthermore, we show that there can be a significant difference, although of small overall magnitude between replicate image datasets (*n* = 3) processed using the same SfM + MVS software, following the same workflow, with a variance in RMSE of up to 1.3, 1.5, and 2.7 cm (excluding one outlier) for “High,” “Medium,” and “Low” quality settings, respectively. We conclude that SfM + MVS software choice does matter, although the differences between products processed using “High” and “Medium” quality settings are of small overall magnitude.

## Graphical abstract

In this manuscript, we show that while centimetric resolution aerial photographic data captured from a low‐flying multirotor drone can deliver new insights into the spatial heterogeneity of an intensively managed grassland sward, there are important, previously neglected, methodological uncertainties. We show that there are significant differences in the quality of the information derived from replicate image datasets and different image‐based modeling software. This understanding is crucial for the development of drone and image‐based modeling workflows if it is to fulfill a role as a new quantitative remote sensing tool to inform management frameworks and species conservation schemes.

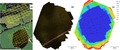

## 1 INTRODUCTION

There is a pressing need within ecology for spatial data that can deliver information about ecosystem functional traits and their dynamics through time. Due to the rapid and at times complex nature of ecosystem dynamics, it is critical to have access to agile, effective, and reproducible methods for capturing key habitat or species traits such as canopy structure. Such data can allow differentiation between early trends and short‐term fluctuations and can also be used for identifying and establishing conservation sites with specific protected features (Fourcade & Öckinger, 2017). An example habitat requiring such information is high‐value temperate grasslands, which are threatened by agricultural intensification (Fritch, Sheridan, Finn, McCormack, & Ó hUallacháin, 2017; Ridding, Redhead, & Pywell, 2015) and climate change (Ibáñez et al., 2013; McCauley, Ribic, Pomara, & Zuckerberg, 2017). Remote sensing techniques have proven their worth in delivering spatio‐temporal data for evaluating ecosystem dynamics across a range of ecosystems (Dalponte, Frizzera, & Gianelle, 2018; Lesak et al., 2011; Luoto, Toivonen, & Heikkinen, 2002; Mori, Tatsumi, & Gustafsson, 2017; Phinn, Menges, Hill, & Stanford, 2000), but in grassland systems there are methodological challenges. Airborne LiDAR‐derived data products potentially provide the best opportunity for gathering fine‐grained measurements describing grassland vegetation structure (Müller et al., 2018), but laser penetration through the canopy can be inconsistent and factors including vegetation canopy density can bias results (Luscombe et al., 2015). Hence, it is not straight forward to determine whether the signals originate from the canopy and soil surface, or if the signal represents something in between (Bretar & Chehata, 2007; Yang, Ni‐Meister, & Lee, 2010). Consequently, new techniques are needed for delivering operational, cost‐effective measurements describing the spatial distribution of fine‐grained canopy structure in such ecosystems (Forsmoo, Anderson, Macleod, Wilkinson, & Brazier, 2018).

Structure from Motion (SfM) and Multi‐View Stereo (MVS) is a rapidly evolving technique for measuring surface structure in ecology (Dandois and Ellis, 2010; Forsmoo et al., 2018; Lucieer, Robinson, Turner, Harwin, & Kelcey, 2012; Remondino, Barazzetti, Nex, Scaioni, & Sarazzi, 2011; Tao, Lei, & Mooney, 2011; Turner, Lucieer, & Watson, 2012; Verhoeven & Vermeulen, 2016), and arguably, this offers the only realistic alternative to LiDAR for measuring the canopy structure of low‐sward systems (Forsmoo et al., 2018). The emergence of SfM + MVS‐based data analysis approaches has been complemented in recent times by an upsurge in drone‐based environmental monitoring (Anderson & Gaston, 2013). The two approaches combined offer a means of executing a workflow for low cost and frequent capture of fine‐grained data to generate surface structural models, including digital surface models (DSMs) from which vegetation height metrics may be obtained (Dandois, Olano, & Ellis, 2015; Forsmoo et al., 2018).

The quality of drone and SfM + MVS‐based models depends on a range of factors including type of camera used and flying speed and altitude, with work by O'Connor, Smith, and James (2017) showing how varying camera settings can impact SfM + MVS‐based data products. There are also issues of methodological‐based uncertainty to consider, for example the impact of lighting conditions and image overlap on resultant model quality (Dandois et al., 2015; James, Robson, & Smith, 2017). Additionally, there are now a great number of commercial or free and/or open‐source SfM + MVS software options that are available for researchers and stakeholders to use. Table 1 summarizes those softwares that are available, but restricts the list to include only those with GPS‐based capabilities, since these can be used to generate spatially meaningful mapping products. From a user's perspective, it is difficult to evaluate which of these software options is optimal, because there is a lack of comparative work that evaluates the products against a consistent baseline. This is particularly true with respect to proprietary SfM + MVS‐based software, where there is little to no information on the algorithms used (Smith, Carrivick, & Quincey, 2016; Verhoeven et al., 2015). Indeed, Fraser and Congalton (2018) call for more analysis on SfM + MVS‐based approaches. Hence, there is a need to quantify the influence of software on data quality, and yet to our knowledge, there have been no statistically robust investigations of this type. This makes it challenging to attribute differences in results to variations in the SfM + MVS‐based method (e.g., software used). This problem limits the quantitative understanding of change in ecosystems surveyed using an SfM + MVS‐based workflow, which is what this paper sets out to test.

**Table 1:** Examples of SfM + MVS‐based software options available for researchers (accessed December 2018)

Software	Link
*Agisoft Photoscan Pro*	http://www.agisoft.com/
*Pix4D*	https://www.pix4d.com/
*3DFlow Zephyr Pro*	https://www.3dflow.net/3df-zephyr-pro-3d-models-from-photos/
*MICMAC*	https://github.com/micmacIGN/micmac
*GRAPHOS*	https://github.com/itos3d/GRAPHOS
*Autodesk Recap*	https://www.autodesk.com/products/recap/overview
*ESRI Drone2Map*	https://www.esri.com/en-us/arcgis/products/drone2map/overview
*SURE*	http://www.ifp.uni-stuttgart.de/publications/software/sure/index.en.html
*Photomodeler Premium*	https://www.photomodeler.com/index.html
*RealityCapture*	https://www.capturingreality.com/

The experiment described in this manuscript sought to determine the influence of SfM + MVS‐based software used to process aerial photographs captured from a low‐flying multirotor drone, over a low sward, intensively managed grassland system. The experiment quantifies the extent to which derived sward height measurements can be replicated and thus facilitates the adoption of SfM + MVS‐based workflows for land management frameworks and conservation schemes. We explored and evaluated this problem by quantifying the influence of the choice of SfM + MVS software and replicate image acquisition workflows. Specifically, the following hypotheses were tested:

1. Three independently captured, replicate image datasets taken over the same field, but from different drone flights (where the drone followed the same preprogrammed flightplan), and processed using the same SfM + MVS workflow can produce significantly different digital surface models (DSMs).
2. The vertical error in SfM + MVS‐derived DSMs varies significantly between different SfM + MVS software when the same image set from the same flight is processed.
3. The vertical error in SfM + MVS‐derived DSMs decreases with increasing computational cost.
4. The costs of different SfM + MVS software approaches are not significantly different in terms of learning, processing, and analytical time as well as financial cost to the user.

## 2 MATERIALS AND METHODS

### 2.1 Study area

The study area was a single agricultural field (8,059 m^2^) located on a grazed, organic dairy farm in Cornwall, southwest England (50°12′09.5″N 5°09′28.4″W, 90 m above mean sea level) with a surface cover of *Lolium perenne* (perennial ryegrass) and *Trifolium pratense* (red clover). The site included a 25 × 20 m patch of set‐aside, unmanaged grassland. The site was chosen because there is a need to understand short sward ecosystems where it is difficult to derive high quality DSMs (Forsmoo et al., 2018; Zahawi et al., 2015). The site was gently sloping with a maximum elevation of 90.8 m (HAMSL) and minimum elevation of 86.8 m (HAMSL).

### 2.2 In situ sward height and topographic validation data

In situ data were collected using a centimeter precision and accuracy differential GPS (DGPS; a Leica GS08plus base and rover GNSS system). Over 2 days, and immediately following the drone flight acquisitions, 236 DGPS data points were collected inside the area covered by the SfM + MVS DSM (6,800 m^2^). The DGPS points were collected across the full spatial extent of the field using a systematic survey pattern, walking along near‐linear transects where the direction and sampling frequency were varied according to the perceived degree of topographic heterogeneity. Data points were collected more frequently where the perceived topographic heterogeneity was greater, that is, where breaks in slope occurred. In addition to the DGPS data points, sward height measurements were collected using a drop disk (Stewart, Bourn, & Thomas, 2001; Waring, 1992) method at the DGPS data point locations as outlined in Forsmoo et al. (2018).

### 2.3 Drone aerial photography survey

**Figure 1:**
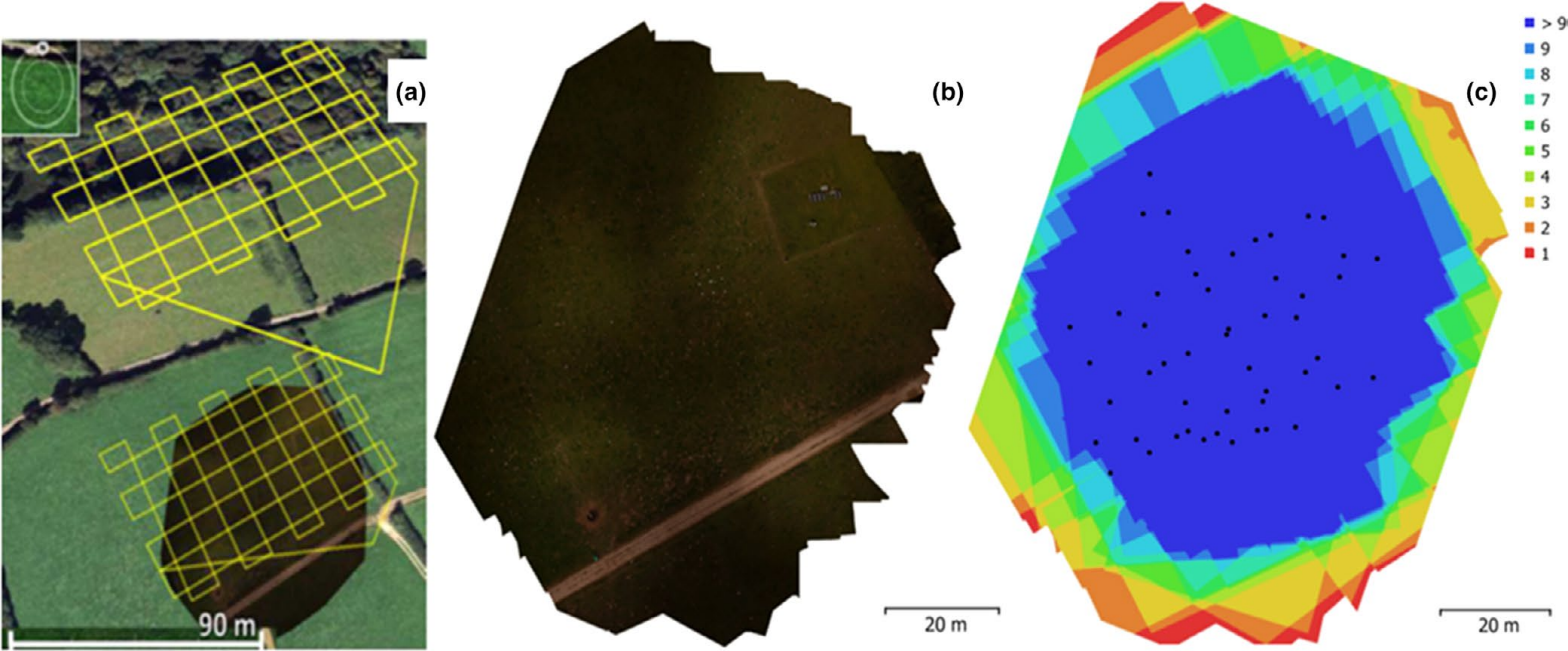
(a) Waypointed route as planned in Mission Planner (ver. 1.3.38), (b) orthomosaic depicting the field site, (c) amount of overlap between the images used in this study, seen over the extent of the field site, where black dots indicate camera trigger locations, and red and white dots indicate the location of the GNSS data points

A small multirotor drone (3D Robotics Iris) was used to obtain aerial photographic data of the field on 21 June, 2016 when the grass was in a period of active growth. The (mean) wind speed during the flight was 2 ms^−1^. The 3DR Iris was chosen due to its low cost (US$400), good reputation regarding flight stability and low rate of mechanical and electrical failures, lightweight construction (1,020 g take off‐weight), and ease of use. A multirotor drone was chosen over a fixed wing drone due to the small area covered and to reduce photographic motion blur. A fixed, prime lens consumer‐grade digital camera (Ricoh GR II) was used to capture the images, and a Pixhawk autopilot guided the drone along a waypointed route (see Figure 1a–c). A more detailed description of the camera settings is outlined in Forsmoo et al. (2018).

Mission Planner (ver. 1.3.38) software was used to prepare the flight. A cross‐stitch lawnmower flight pattern was chosen (Figure 1c), with 70%/70% side/forward overlap in each of the two directions of the grid. Fourteen georeferenced high contrast markers were dispersed throughout the study area using a cluster of ten in the center of the scene and four in two of the opposite edges of the scene, following recommendations by Cunliffe, Brazier, and Anderson (2016). The georeferenced markers were used to convert the SfM + MVS generated DSMs from a relative coordinate system to British National Grid (BNG36)—these markers were surveyed in terms of their *x,y,z* position using the DGPS. Flying at a height of 50 m, the drone produced image data with a ground sampling distance (GSD) of between 0.52 and 0.60 cm. The survey was repeated three times using exactly the same parameters and following the same flight plan each time, to allow replication and therefore reproducibility of the approach to be understood (following recommendations of Dandois et al., 2015). The three replicate image datasets were captured in the time span of an hour, ensuring confidence that there was no measurable change in the variables being measured (land surface height and sward height) between the three flights.

### 2.4 SfM + MVS workflow

An SfM + MVS workflow applies computer vision algorithms to images with a high degree of overlap to place the images taken in 3D space (Forsmoo et al., 2018; Remondino, Nocerino, Toschi, & Menna, 2017; Rupnik, Daakir, & Pierrot Deseilligny, 2017; Smith et al., 2016; Verhoeven & Vermeulen, 2016). These computer vision algorithms are implemented in numerous ways depending upon software choice, where the SfM + MVS workflows range from semi‐automatic, where each step such as identification of key points and camera calibration is called separately, to a fully automated workflow. Four state‐of‐the‐art^1^ examples of SfM + MVS software currently available were tested here, chosen because they represent various commercial options at different price points (Agisoft Photoscan, Pix4D, 3DFlow Aerial) to a free‐to‐use and an open‐source option (MICMAC). To reduce the influence of the “human factor,” the same location (pixel coordinates) of georeferenced high contrast markers in the aerial 2D images was used across the four different software. The citations given alongside indicate other literature examples that have utilized these software in ecology research:

1. 3DFlow Zephyr Aerial (little evidence of use in ecology, though widely used in urban environments, e.g., Vassena & Clerici, 2018; Peel, Luo, Cohn, & Fuentes, 2018; Azzola, Cardaci, Mirabella Roberti, & Nannei, 2019).
2. Agisoft Photoscan (Cunliffe et al., 2016; Dandois et al., 2015; Hoffmann et al., 2015; Javernick, Brasington, & Caruso, 2014; Lucieer, Turner, King, & Robinson, 2014; Obanawa & Hayakawa, 2015).
3. Pix4D (Magtalas, Aves, & Blanco, 2016; Ouédraogo, Degré, Debouche, & Lisein, 2014; Raeva, Filipova, & Filipov, 2016).
4. MICMAC (Forsmoo et al., 2018; Lisein, Pierrot‐Deseilligny, Bonnet, & Lejeune, 2013; Ouédraogo et al., 2014; Tournadre, Pierrot‐Deseilligny, & Faure, 2014; Tournadre, Pierrot‐Deseilligny, & Faure, 2015).

The SfM + MVS software compared is presented in Table 2. Several criteria describing ease of use and cost are presented.

**Table 2:** Overview of the software used in the study

Software	Documentation	Support/community	Under development	CPU time “High”/“Medium”/“Low” (min)^a^
*3DFlow Aerial (Ver 3.700)*	Yes, including algorithms used	Email and forum	Yes, last release: April 2019	891/170/61
*MICMAC (Ver. 1.0.beta11‐459)*	Yes, including algorithms used.	Forum	Yes, last update: February 2019	113/29/24
*PhotoScan PRO (1.4.1)*	Yes, excluding algorithms used.	Email and forum	Yes, last update: March 2019	663/64/31
*Pix4DMapper (4.1.25)*	Yes, excluding algorithms used.	Email and forum	Yes, last update: March 2019	60/7/2

Note

Information accessed 28 April 2019, and is subject to change.

a Workstation: Consumer‐grade desktop (AMD Ryzen 1800x CPU, 16GB DDR4 RAM, AMD RX 570 GPU)

**Figure 2:**
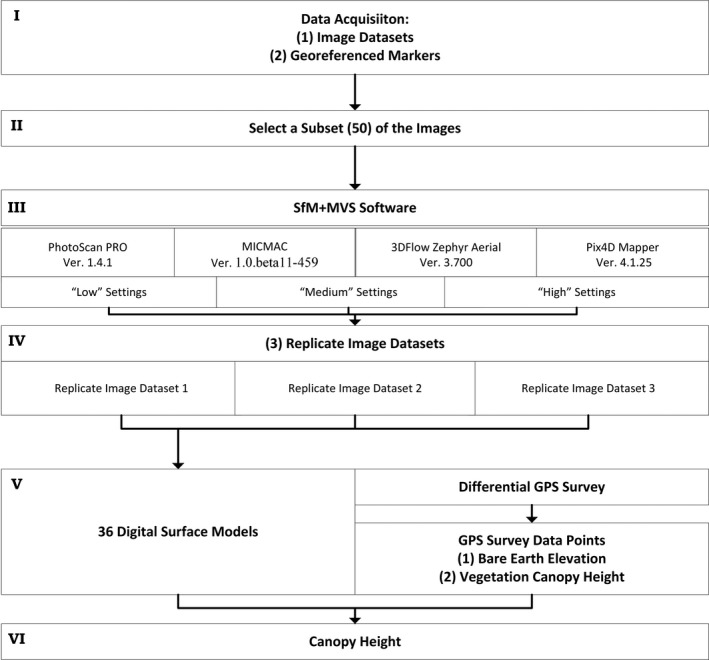
Workflow outline. A typical SfM + MVS workflow, the workflow utilized in this study, is outlined. The major steps in terms of computational cost or labor intensity are as follows: (I) aerial images are collected using a consumer‐grade drone along waypointed route, (V) generate a DSM in an absolute coordinate system (e.g., BNG36), (VI) utilize the SfM + MVS DSM and in situ collected DTM data points to calculate the sward canopy height

Figure 2 presents an overview of the critical methodological steps followed for the comparison work undertaken here, including data acquisition and the drone and SfM + MVS‐based workflow.

To reduce the computational cost of generating 36 SfM + MVS DSMs, a subset of 50 images were selected from the image datasets. The subset of images (*n* = 50) was used for all software (*n* = 4). The selection of a subset of images was undertaken using the MICMAC tool OriConvert, which used a specified image as the master image, and selects the specified number of neighboring images based on the coordinates of the geotagged images. The master image was selected by choosing the image covering the same scene from the same angle in the three replicate image datasets, respectively.

Each of the proprietary software (Pix4D, 3DFlow, and Photoscan) methodologies was learnt in <3 days (Table 1). MICMAC was significantly more difficult to learn—and took the lead author of this paper approximately 30 days, though the exact time required does depend on user experience and expertise. The three main factors contributing to MICMAC's relatively steep learning curve were as follows:

1. MICMAC is compiled from source.
2. The MICMAC workflow used in this study was not detailed in the MICMAC manual.
3. MICMAC consists of numerous modules that can be combined in several ways.

This learning curve can be compared to the three proprietary software (Pix4D, 3DFlow, and Photoscan)—where the SfM + MVS workflow is predetermined, and most of the steps used commonly are automatically carried out via drop‐down menus. The greatest user‐based learning involved with the three proprietary software was how to convert the SfM + MVS model from a relative coordinate system to an absolute coordinate system, a step in the process which differs between software. The MICMAC application took the lead author of this paper approximately 30 days to learn.

In terms of computational cost, three different processing workflows (“High,” “Medium,” and “Low”) were identified for each software (*n* = 4). These settings were used for each replicate image dataset (*n* = 3) to explore how accuracy depends on theoretical grade of desktop workstation or server the user has access to (see Table 3).

**Table 3:** The settings and version used for each of the software, respectively

Software	3DFlow Zephyr Aerial	Photoscan PRO	Pix4DMapper	MICMAC
Settings (“High”)/Full sized images	Matching type: accurate Matching stage depth: full Discretization: very high Discretization: very high	Accuracy: highest Quality: ultra high Depth filtering: mild	Keypoints image scale: full Aerial grid Geometrically verified matching	Tapioca file ‐1 Tapas Radial Extended + Figee Malt Ortho SzW = 1 ZoomF = 1
Settings (“Medium”)/Downscaled images (50%)	Matching type: accurate Matching stage depth: high Discretization: very high Resolution: ½ original size	Accuracy: high Quality: high Depth filtering: mild	Keypoints image scale: ½ original size Aerial grid Geometrically verified matching	Tapioca file 2464 Tapas Radial Extended + Figee Malt Ortho SzW = 1 ZoomF = 2
Settings (“Low”)/Downscaled images (25%)	Matching type: accurate Matching stage depth: high Discretization: high Resolution: ¼ original size	Accuracy: “Medium” Quality: “Medium” filtering: mild	Keypoints image scale: ¼ original size Aerial grid Geometrically verified matching	Tapioca file 1232 Tapas Radial Extended + Figee Malt Ortho SzW = 1 ZoomF = 2
Version	3.700	1.4.1	4.1.25	Ver. 1.0.beta11−459

### 2.5 DSM generation

Sward height validation points located in edges with poor image overlap (*n* < 3) and/or which were not covered by either of the dense SfM + MVS point clouds were removed. This left 228 sward height validation points for further analysis. The extent of the dense point cloud was divided into 1.2 × 1.2 cm grids. The maximum elevation of each 1.2 × 1.2 cm cell was used to generate a continuous DSM from the dense SfM + MVS point cloud. A 1.2 × 1.2 cm grid DSM was chosen to cover ca. twice the footprint as the image data. This operation was undertaken using the free and open‐source CloudCompare software (ver. 2.9.1).

### 2.6 Comparison of SfM photogrammetric outputs with ground validation data

To quantify the quality of the DSM generated using an SfM + MVS workflow, the SfM + MVS model was compared to sward height ground validation data. The elevation was extracted at the locations where the DGPS (soil surface elevation and sward height) was measured. This was done for all the points (*n* = 228) using the GIS software, ArcMap (ver. 10.2.2).

The measures of quality included in this study were (a) Root Mean Square Error (RMSE) and (b) correlation coefficient (*R* ^2^) between validation sward height and the sward height measured using the proposed SfM + MVS workflow. These measures were computed in MATLAB (ver. 2016b).

To test for significant difference between results, a two‐sided, paired *t* test was used with an alpha value of 0.05. This was carried out using MATLAB 2016b. More specifically, the following were tested for significance:

1. Is there a significant difference between results from different software (*n* = 4) when using the same image dataset and the same ground control points?
2. Is there a significant difference between replicate image datasets (*n* = 3) processed using the same software and workflow?
3. Is there a significant difference between the combined results (software *n* = 4) for replicate image datasets (*n* = 3)?

### 2.7 Change detection with M3C2

The Multiscale Model to Model Cloud Comparison (M3C2) algorithm detailed in Lague, Brodu, and Leroux (2013) allows for robust comparison of fine‐grain points clouds from complex natural environments (James, Robson, & Smith, 2017). Specifically, M3C2 works directly with the point cloud—whereas previous methods such as DEM of difference (DoD) require rasterized data which do not allow point‐to‐point‐based properties to be taken into consideration. M3C2 therefore has the capacity to accurately capture mean surface change in noisy datasets/environments. Additionally, M3C2 offers a key advantage in the ability to estimate local confidence intervals which enables calculation of significant change across space and time. Herein, M3C2‐based analyses were applied to pairs of point clouds (*n* = 54) to evaluate spatial differences in SfM + MVS‐derived DSMs between (a) replicate image datasets and (b) software.

To understand the rationale for using M3C2, one must understand how it works. In short, M3C2 consists of two steps: First, for each point cloud a plane is fitted to the points within the radius *D*/2 of point *i*, which enables the calculation of a normal vector. Secondly, the normal vector is used to calculate the distance between two clouds by projecting point *i* onto each of the clouds at the projection scale *d*. This makes it possible to estimate the average position of each cloud (*i*1 and *i*2) around point *i*. A measure of the local distance between the two clouds is defined as the distance between *i*1 and *i*2. More specifically, this is achieved by defining a cylinder of radius *d*/2 with the axis through point *i*, and which is oriented along the normal vector. Where each of the two point clouds intercepts the cylinder, there will be two subset of points (one for each point cloud), *n*1 and *n*2. Projecting *n*1 and *n*2 onto the axis of the cylinder generates two sets of distance distributions. The mean of these distributions is used to approximate the local surface roughness. The local surface roughness and subset of points, *n*1 and *n*2, in turn allow for the calculation of a local confidence interval (Barnhart & Crosby, 2013; Lague et al., 2013). For a more detailed explanation, see Lague et al. (2013). The M3C2 parameters used herein are based on recommendations by Lague et al. (2013), specifically, normal scale *D* ~ 20 times the (95th percentile) surface roughness (96 cm), projection scale *d* = 10 times the number of points per unit area in the point cloud, subsample = subsampled to 6 cm, or ~5 times the ground sampling distance, as a compromise between computational cost and resolution.

## 3 RESULTS

### 3.1 Overview of field site and drone survey

**Figure 3:**
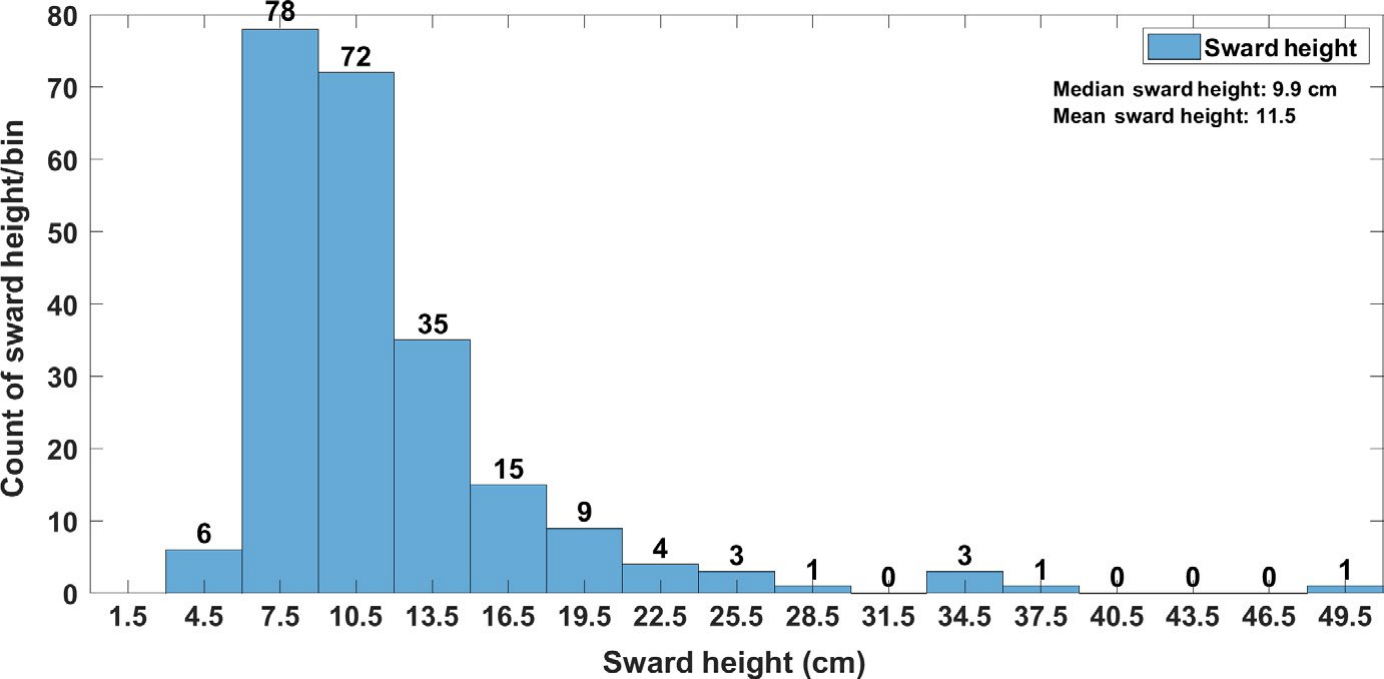
Sward height distribution of in situ validation measurements of sward height

Over 90% of the field site was covered by a high degree of image overlap with at least three images per point, but with a central area of interest coinciding with the field validation points where overlap was consistently very high (see Figure 1). The remaining ~10% where image overlap was <3 images per point was excluded from the analysis. In situ measurements on the day of the drone flight showed that the mean canopy height was 11.5 cm (min: 4.9 cm, max: 48.4 cm; Figure 3). 

### 3.2 Reproducibility with computational cost

**Figure 4:**
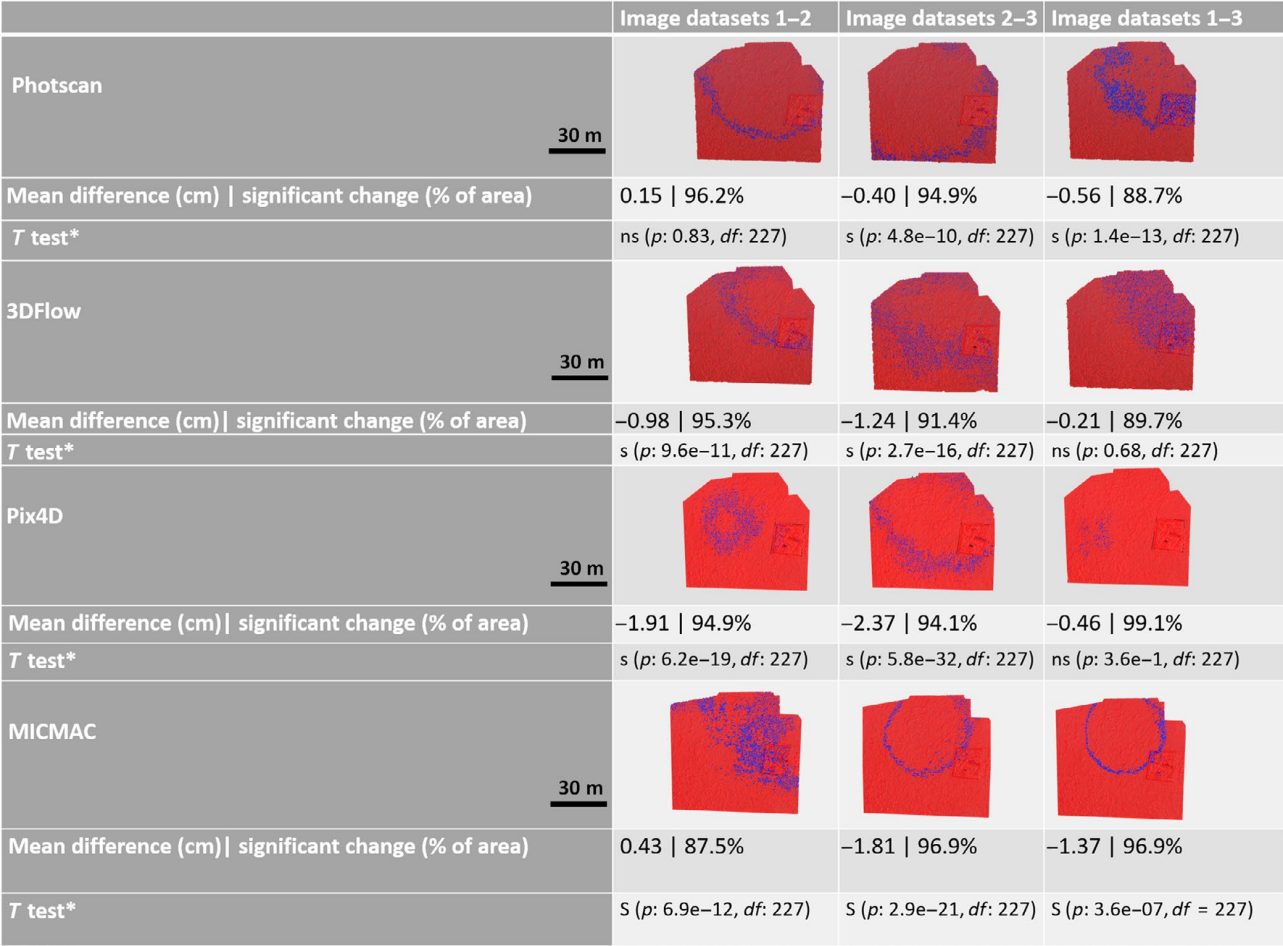
Spatial distribution of significant changes between replicate image datasets (*n* = 3) for four software (Photoscan, 3DFlow, Pix4D, and MICMAC) at “High” quality settings, respectively. *(ns = not significant, s = significant)

To understand the robustness of the software better, the significant differences between the resulting dense point clouds for each of the three replicate image datasets were computed using the M3C2 method (Lague et al., 2013). This was carried out for each software (*n* = 4) using CloudCompare (ver. 2.9.1; see Figures 4, S1 and S2, Appendix S1).

#### 3.2.1 Replicate image datasets

**Figure 5:**
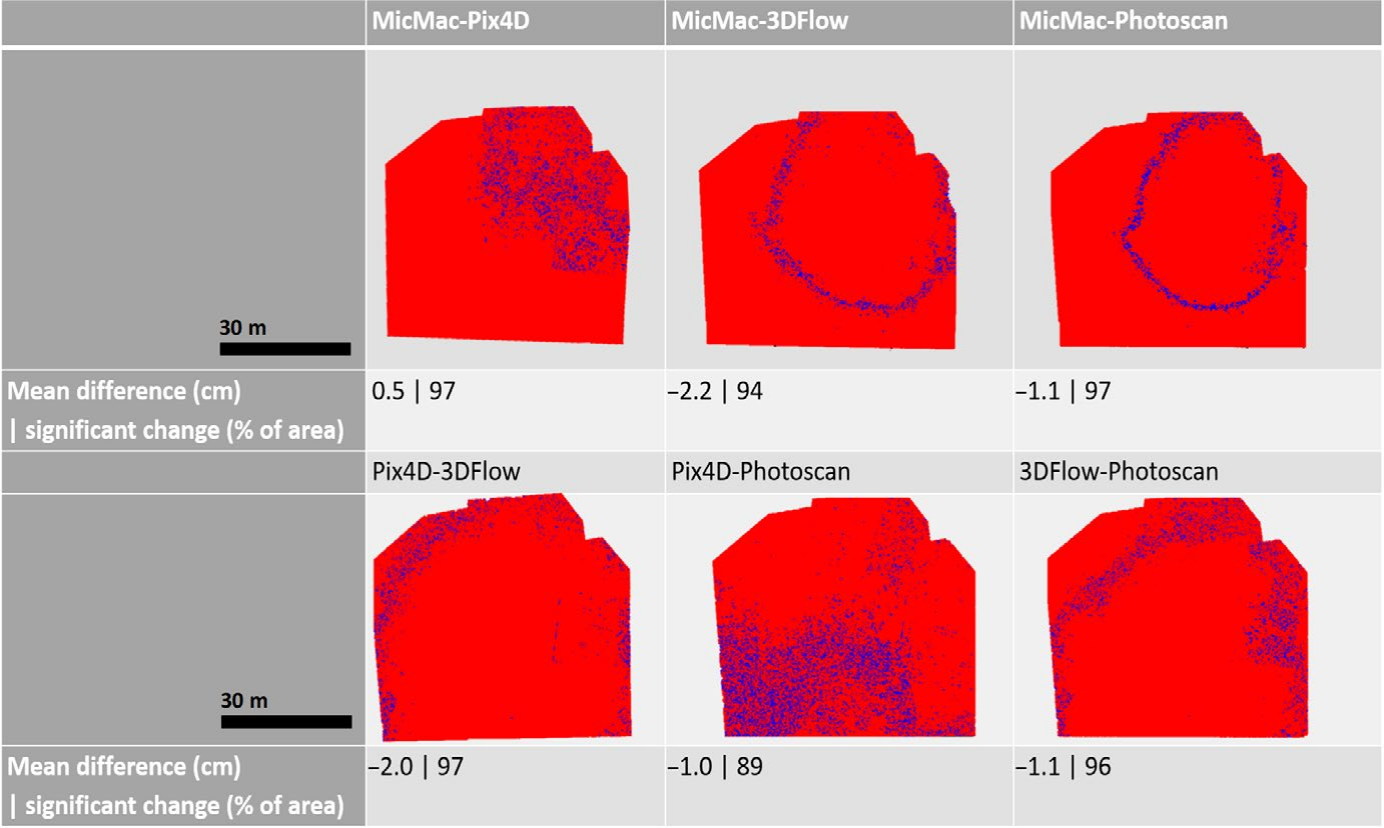
Boxplot of the RMSE of the SfM + MVS‐derived sward heights generated using the three replicate image datasets, compared to sward height validation data. The data on the x‐axis are labeled according to replicate image dataset (1–3), and validation data (sward height). (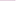) indicates the median (RMSE), (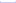, lower and upper) represents the 25th and 75th percentiles, respectively, and (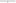) shows the minimum and maximum data point value (Matlab, 2017)

A boxplot of the RMSE for Pix4D, Photoscan, 3DFlow, and MICMAC for each of the three image datasets with “High” quality settings is shown in Figure 5. The median RMSE of the SfM + MVS‐derived sward height is consistently reduced when using higher quality settings when compared to sward height validation data (*n* = 228; see Figures 5 and S3, Appendix S1).

To determine if there is a significant difference, overall, in derived height measurements between replicate image datasets, a paired *t* test was used. It was found that there was a statistically significant difference between the SfM + MVS‐derived DSMs produced between each of the three replicate image datasets (first–second, first–third, and second–third), for each of the three quality settings (“High,” “Medium,” and “Low”; see Table 4).

**Table 4:** Using a paired *t* test, differences between the SfM + MVS‐derived DSMs produced using replicate image datasets were tested for significance

Paired *t* test: *df*: 911; alpha: 0.05		First–second	First–third	Second–third
“High” settings	p:	3.4e−33	1.3e−69	5.4e−08
“Medium” settings		4.8–33	1.3e−71	3.3e−18
“Low” settings		1.9e−19	2.6e−17	1.9e−18

Note

DSM height measurements from each software (*n* = 4) were combined, which was then compared between the three replicate image datasets (first–second, first–third, and second–third).

### 3.3 Reproducibility across software

**Figure 6:**
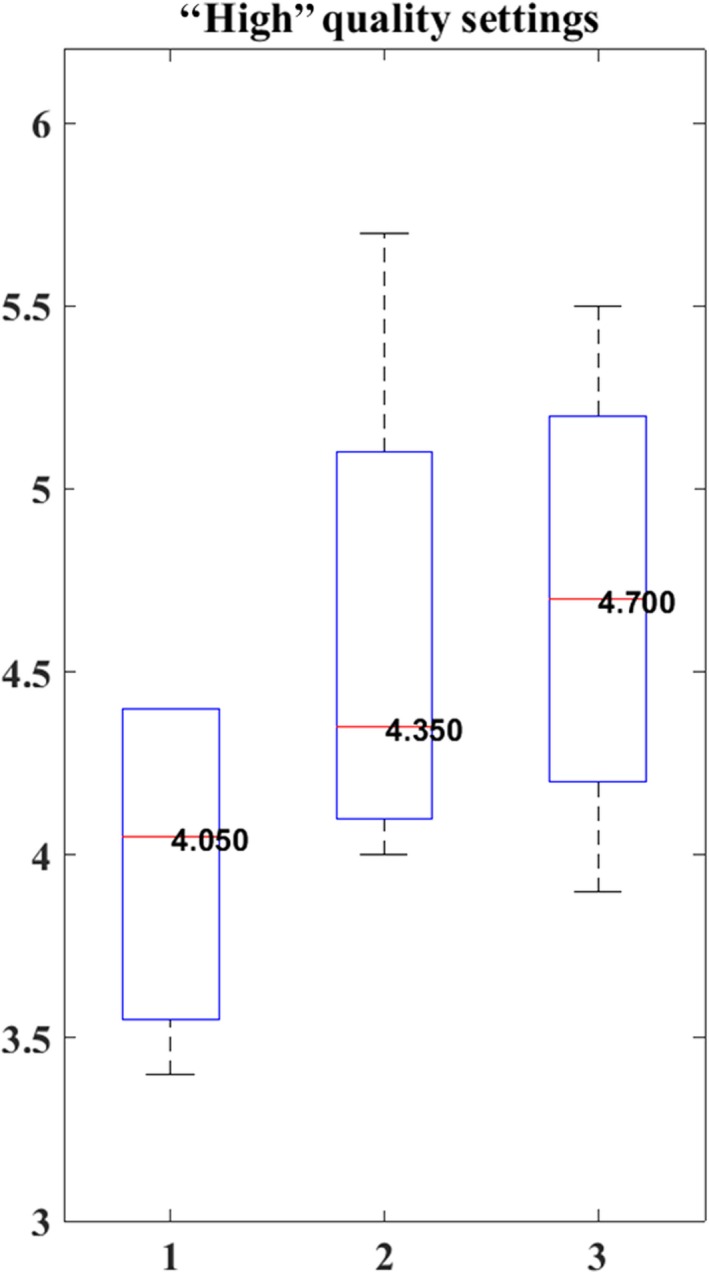
Spatial distribution of significant changes between software (*n* = 4) for one replicate image dataset (#2) and “High” quality settings, respectively

To understand the robustness of SfM‐MVS‐based workflows better, the significant differences between the resulting dense point clouds were computed using the M3C2 method (Lague et al., 2013). This was carried out between each of the software (*n* = 4) and the second replicate image dataset using CloudCompare (ver. 2.9.1; see Figures 6, S3 and S4, Appendix S1).

#### 3.3.1 Key statistics

The number of points per unit area is not necessarily a robust indicator of quality. However, it can provide a rough gauge for the quality of processing settings used—and conversely what one can expect following the workflow outlined herein. Image residual (pixels) is the mean local error in image alignment, as estimated by the bundle adjustment (Bogunovic et al., 2014; Forsmoo et al., 2018; James, Robson, & Smith, 2017). GCP residuals show the difference between measured coordinates and the corresponding coordinates within the SfM + MVS‐derived 3D model (James, Robson, d'Oleire‐Oltmanns, & Niethammer, 2017). As a rough guideline, one tries to aim for an image residual below half a pixel, and a GCP residual below 2 cm, though the requirements differ between use cases.

##### 3.3.1.1 High settings

Table 5 allows comparison between software and, in particular, elucidates the identification of absolute and relative difference between replicate image datasets. This is for the “High” quality settings.

**Table 5:**
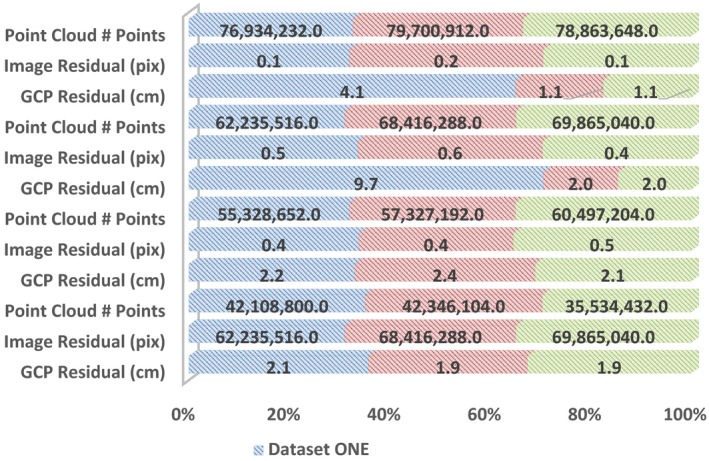
Overview of three variables of interest: (i) point cloud # points, (ii) image residual, and (iii) GCP residual for each software (*n* = 4) and replicate image dataset (*n* = 3) using “High” quality settings

### 3.4 Replicated independent image datasets and different SfM software produce significantly different DSMs

**Figure 7:**
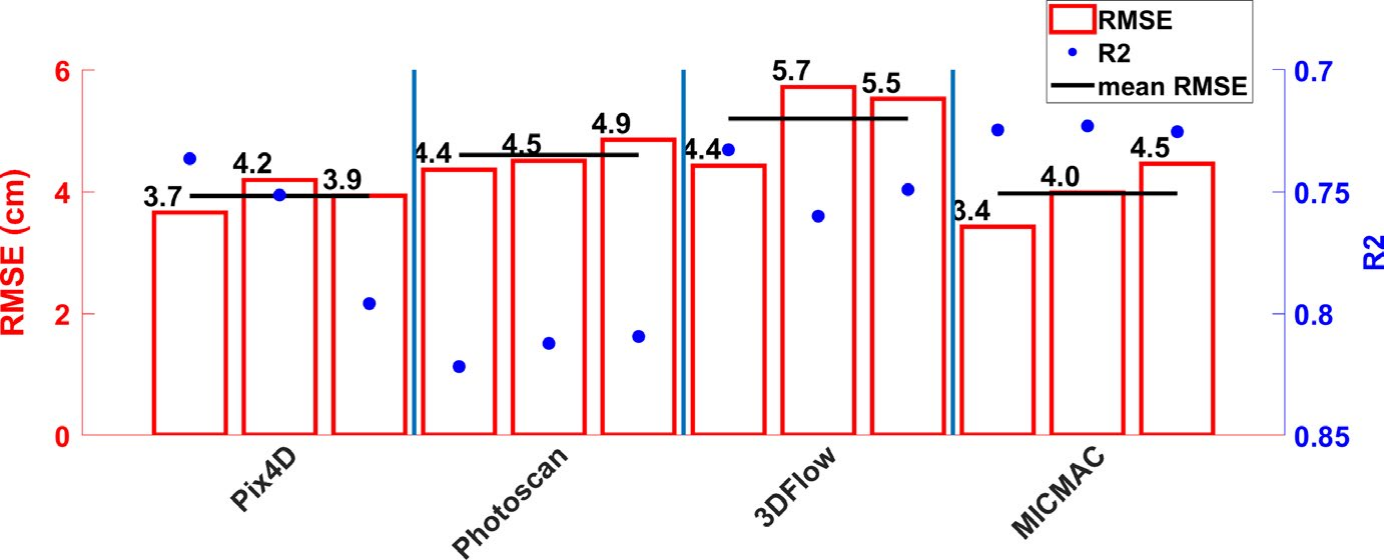
“High” settings. The Root Mean Square Error (m, RMSE) (bar) and *R* ^2^ (axis reversed) (dot) for each of the SfM + MVS‐derived DSMs, for each of the three replicate image datasets. The black line indicates the mean RMSE for each of the SfM + MVS software, respectively. The replicate image datasets are in order—1 to 3, from left to right, for each of the SfM + MVS software tested

Sward height measurements derived from an SfM + MVS workflow were compared to in situ validation sward height measurements (see Figure 6). The SfM + MVS‐derived measurements are compared in terms of RMSE and *R* ^2^. The RMSE ranged from 3.4 cm to 5.7 cm for MICMAC and 3DFlow, respectively, seen over the three replicate image datasets. The correlation coefficient (*R* ^2^) was calculated as the correlation between validation sward height and the sward height measured using the proposed SfM + MVS workflow. Using a paired *t* test, it was found that there was a statistically significant difference between the model with lowest RMSE and the model with the highest RMSE for the first, second, and third replicate image datasets, respectively, using “High” quality settings. While improvements are significant in statistical terms, the differences, given the magnitude, are minimally important in practice. The replicate image datasets are in order—1 to 3, from left to right (see Figure 7).

### 3.5 Is there an important difference in financial cost between software?

To allow users to quantify software differences in terms of financial cost, customizability, and ease of use, a simple matrix was developed. The first step (see Table 6) quantifies the different software in terms of (a) customizability, (b) financial cost, (c) CPU time, (d) ease of use, and (e) range of data products ranked between 1 and 4 (the higher the better. In case of tie, the same rank is given). Customizability refers to the extent a user can modify the core settings of the software and/or the type of analysis carried out. For example, in Photoscan and Pix4D a user is restricted to a limited number of key parameters (number of tie points, number of key points etc.), whereas in 3DFlow and MICMAC, a user can often adjust more than 20 different parameters at each step in the processing pipeline. MICMAC gets the higher rank, though, for its flexible processing pipeline, where different modules can be combined in several different ways depending on the user's needs. Also, worth pointing out that MICMAC gets a rank of 2 in ease of use/support for the fact that since this study was started, articles such as Rupnik et al. (2017) have been published, which simplifies the learning process.

**Table 6:** Each software has been given a value between 1 and 4 for each of the five categories deemed to be of importance

	Customizability/flexibility	Financial cost	CPU time/computational cost	Ease of use/support	Range of data products
3DFlow	3	2	1	4	4
MICMAC	4	4	3	2	2
Photoscan	1	3	2	4	4
Pix4D	1	1	4	4	4
	9	10	10	14	14

Note

The value given is, where possible, based on actual data such as CPU time in minutes and acquisition cost of software (as of 08/2018).

By dividing the score for each software (*n* = 4) for each category (*n* = 5) by the total score for each category, each score can be normalized (see Table 7).

**Table 7:** The score for each software (*n* = 4) for each category (*n* = 5) is divided by the total score for each category

	Customizability/flexibility	Financial cost	CPU time/computational cost	Ease of use/support	Range of data products
3DFlow	3/9 = 0.3333	2/10 = 0.2	1/10 = 0.1	4/14 = 0.2857	4/14 = 0.2857
MICMAC	4/9 = 0.4444	4/10 = 0.4	3/10 = 0.3	2/14 = 0.1429	2/14 = 0.1429
Photoscan	1/9 = 0.1111	3/10 = 0.3	2/10 = 0.2	4/14 = 0.2857	4/14 = 0.2857
Pix4D	1/9 = 0.1111	1/10 = 0.1	4/10 = 0.4	4/14 = 0.2857	4/14 = 0.2857

Note

This yields a normalized score for each category and software.

With each score normalized, the user can rank the five different categories in terms of their relative importance. The normalized value is multiplied with the user‐defined rank which can be adjusted depending on the project (the example values chosen below are for the study detailed herein). The score for each software and category can then be added together. Table 8 outlines an example.

**Table 8:** The normalized score for each category is multiplied by a user‐defined rank which is based on the five different categories relative importance

User‐defined rank of importance	5	4	3	2	1	
	Customizability/flexibility	Financial cost	CPU time/computational cost	Ease of use/support	Range of data products	
3DFlow	0.3333 × 5 = 1.6667	0.2 × 4 = 0.8	0.1 × 3 = 0.3	0.2857 × 2 = 0.5714	0.2857 × 1 = 0.2857	3.6
MICMAC	0.4444 × 5 = 2.222	0.4 × 4 = 1.6	0.3 × 3 = 0.9	0.1429 × 2 = 0.2857	0.1429 × 1 = 0.1429	5.2
Photoscan	0.1111 × 5 = 0.5556	0.3 × 4 = 1.2	0.2 × 3 = 0.6	0.2857 × 2 = 0.5714	0.2857 × 1 = 0.2857	3.2
Pix4D	0.1111 × 5 = 0.5556	0.1 × 4 = 0.4	0.4 × 3 = 1.2	0.2857 × 2 = 0.5714	0.2857 × 1 = 0.2857	3.0

Note

The score for each software and category can then be added together.

## 4 DISCUSSION

### 4.1 H1. (1) Replicated independent image datasets can produce significantly different DSMs

We tested whether replicated, proximal image datasets processed using the same workflow produced statistically different topographic models. In order to test this, we collected three replicate image datasets and analyzed them using three different quality settings (“High,” “Medium,” and “Low”). As can be seen in Tables 4 and 5 and Figures 5 and 7 (see also Tables S1 and S2 and Figures S5–S7, Appendix S1), we demonstrated that the above hypothesis has been statistically proven. That is, there is a statistically significant (*p* < 0.05) difference between each of the three replicate image datasets processed using the same workflow, including SfM + MVS software, with “High,” “Medium,” and “Low” settings, respectively (see Table 4). This result is something that all researchers should consider for their particular application, as the true difference could be larger in more heterogeneous systems, with a greater range of vegetation cover and more variable canopy height, for example. Reproducibility of a method is key to be able to attribute detected changes to actual changes within the system of concern, and not artificial differences over time introduced by the methodological approach. To address the variance between replicate image datasets processed using an SfM + MVS workflow, we suggest to incorporate replicate image datasets in an SfM + MVS workflow. This is something that has already been outlined as an important consideration by Dandois et al. (2015) who collected five replicate image datasets and used the average of the replicate image datasets for further analysis. However, most studies to date ignore and do not acknowledge reproducibility limitations of an SfM + MVS workflow. As such, the implications of findings of many studies (Hugenholtz et al., 2013; Mancini et al., 2013; Obanawa & Hayakawa, 2015; Ouédraogo et al., 2014; Tonkin, Midgley, Graham, & Labadz, 2014; Wang et al., 2014) are limited as the conclusions are based on a single SfM + MVS model. Further work needs to be carried out to find the optimal number of replicate image datasets to describe potential variance and to find a compromise between reproducibility and computational cost.

#### 4.1.1 M3C2 analysis

The M3C2 analysis suggests two things: (a) that there are (systematic) patterns in the data and (b) that there are relatively few points/areas that are statistically similar across replicate image datasets. While part of this probably can be attributed to vegetation—as the algorithm was developed for scenes with bare soil, it is important to point out that potentially adverse effects associated with vegetation can be minimized with the appropriate choice of constants (Lague et al., 2013). Additionally, this is a cloud‐to‐cloud comparison in an environment that is known to have undergone no physical change in between data collections. Hence, even though the vegetation complicates the analysis, it can in this case be treated as a fixed, albeit complex surface, with fine‐grain topographic patterns. Therefore, we would argue there is still validity to the patterns apparent in the M3C2 analysis.

Systematic patterns in the accuracy analysis of a SfM‐MVS‐derived DSM can be due to vegetation patterns, ground control point distribution, and/or the camera lens calibration model. The predominantly circular patterns present in the data presented in this study do not conform with either the vegetation pattern or the location and distribution of ground control points. Hence, it is likely that the patterns highlighted in Figure 4 (see also Figures S1 and S2, Appendix S1) are due to insufficiencies in the (internal) camera lens calibration model (James & Robson, 2014). This hypothesis is further supported by the fact that systematic patterns are largely software dependent. Hence, as each software uses a different lens calibration model, it may depict the influence of the camera calibration process. A “poor” camera lens calibration model can be improved by including oblique image data as a complement to the nadir image data (James & Robson, 2014) and/or by calibrating the camera lens distortion model using a separate (high quality) image dataset with convergent viewing angles of a textured 3D object.

In order to address the above issue, a fixed camera mount was used in this study, and this provides a greater range of camera viewing angles than the word nadir suggests. Different viewing angles are present because of platform tilt variations present in a regular multirotor drone flight mission. The amount of tilt will vary with, for example, flight speed, wind speed, platform attitude, position of camera mount, etc. Forsmoo et al. (2018) clearly show that these variations in tilt are enough to achieve centimeter accuracy. Having said that, the data do suggest that the results could (likely) consistently be improved by having included additional oblique image data. Hence, it is important to keep in mind that the results presented herein are representative for a vegetated scene with a limited range of viewing angles, and not necessarily for other scenes and methodological approaches.

*Why are replicates not (statistically valid) replicates?* Differences in quality between replicate image datasets could be due to a range of factors including wind speed, light conditions (Dandois et al., 2015), variations in the location (pixel coordinates) of georeferenced high contrast markers in the aerial 2D images—which influence the *x*,*y* bias of the SfM + MVS‐derived DSM, and robustness of the SfM + MVS software (Dandois et al., 2015; James, Robson, d'Oleire‐Oltmanns, et al., 2017). The influence of wind speed and light conditions was studied in Dandois et al. (2015), and both were found not to exert an important influence on the quality of the SfM + MVS‐derived DSM. Having said that, light conditions influence the image contrast (increased contrast with direct lighting) and shadows—which influence the identification of keypoints in images (Lowe, 2004). However, in this study the replicate image datasets were collected within the time span of an hour, with very similar weather conditions (2–3 m/s mean southerly wind speed, 16.8–17.9°C, cloud cover ~ 30%), so we are confident that the light, temperature, and wind conditions were similar and are thus assumed to have an insignificant effect on the results. Yet it is possible that the light wind blowing at the time of the flight would have caused movement in the blades of grass but this is the only expected change between the three flights. Flying height has been discussed and our choice to fly at 50 m was determined to be the optimal compromise between area coverage and data quality (Dandois et al., 2015; Mesas‐Carrascosa et al., 2016).

The robustness of the software is another potential explanation for the observed variance between the replicate image datasets. Given the difference in variance in RMSE for the replicate image datasets between the software (see Figures 7, S6 and S7, Appendix S1), we argue that it is likely that an important part of the variance is due to the robustness^2^ of the SfM + MVS software. This warrants further studies exploring the aspect of robustness—or sensitivity, of the SfM + MVS software, including how the quality of information derived from the software depends on a combination of methodological workflow (Dandois et al., 2015; Verhoeven, 2017) and the attributes (e.g., vegetation, buildings, homogeneity of textures) in and of the surveyed scene (Furukawa & Hernández, 2015; Mancini et al., 2013; Remondino, Pizzo, Kersten, & Troisi, 2012; Ryan et al., 2015; Turner et al., 2012).

### 4.2 H2. (2) Vertical and horizontal error varies significantly between different SfM + MVS software

We accept this hypothesis demonstrating that the choice of software is an important consideration which may determine the quality of the DSM (see Figures 6, 7, S3, S4, S6 and S7, and Appendix S1). There is a statistically significant (*p* < 0.05) difference between the software with the lowest and highest RMSE compared to in situ validation data, respectively, for each of the replicate image datasets (*n* = 3) and choice of quality settings (*n* = 3).

However, the differences might not be of practical significance. While centimeter differences are often important for change monitoring (Forsmoo et al., 2018; Lucieer et al., 2012) and when modeling processes such as surface runoff based on topographic variability (Mügler et al., 2011; Thompson, Katul, & Porporato, 2010), where small differences can lead to important cumulative biases (Liu et al., 2019; Lucieer et al., 2014), it is important to acknowledge that for some, if not many, purposes measurement uncertainties at the centimeter magnitude are neglectable. In fact, we would argue that these fine‐grain uncertainties highlight exactly why a user would choose drones over aerial or satellite imagery for change detection. However, drone and SfM + MVS‐based data can give a false sense of security due to its ease of application and visual appeal, and software factors may become more important than RMSE differences at the centimeter magnitude. It is indeed also important to acknowledge that the analysis presented herein is from a relatively small and homogenous field site, and a larger and more complex image dataset would likely influence the findings (Colomina & Molina, 2014; Remondino et al., 2012).

### 4.3 H3. (3) The vertical error in SfM + MVS‐derived DSMs decrease with computational cost

We demonstrate (Figures 7, S6 and S7, Appendix S1) that the vertical error, on average, decreases with computational cost. The RMSE of the SfM + MVS‐derived DSM for the three replicate image datasets processed using “High” settings is, on average—seen across the software, lower when compared to when processed with “Medium” and “Low” settings, respectively (see Figures 7, S6 and S7, Appendix S1). Therefore, we can confirm that this (3) hypothesis is true. Figure 4 and Table 5 (and Figures S1, S2 and Tables S1, S2, Appendix S1) suggest that changes to the settings affect software differently. While there is a trend toward increasing image residuals (pixels) with decreasing computational cost, Pix4D rather shows dataset‐specific effects that are exacerbated with decreased computational cost (see Table 5 and Tables S1, S2, Appendix S1).

This result might be expected as the computational cost of the SfM + MVS workflow increases the higher the settings used. Though in three instances (see Figures 7, S6 and S7, Appendix S1), the RMSE did increase with computational cost. There are two hypotheses why this could be the case (3DFlow, 2018):

1. Higher number of keypoints results in a higher chance for false matches in homogeneous areas or in scenes with repeated patterns.
2. Downscaled images can reduce the influence of potential pixel‐level camera and/or image compression distortions.

This finding warrants further exploration as few previous studies have investigated the influence of software settings in general, not to mention in low‐height ecosystems where centimeter differences are important from a relative perspective. Centimeter changes can be on the same order of magnitude as that of low‐height vegetation.

### 4.4 H4. (4) The costs of different SfM+MVS software approaches are not significantly different in terms of learning, processing, and analytical time as well as financial cost to the user

When discussing the cost of a method or software of choice, it is important to consider costs versus benefits, including acquisition cost, the processing time, and hours invested in learning the software. While there were important differences between the software, both in terms of processing time and ease of learning (see Tables 2, 6, 7, 8)—each software has its own advantages and disadvantages. Hence, the recommended software depends on the type and requirements of the application/project in question and the relevant expertise of the user. For example, while a Pix4D license comes at a relatively high financial cost it offers straightforward and seamless integration with a range of camera types, such as the multispectral camera Sequoia and the thermal cameras Zenmuse XT and Flir VUE Pro. MICMAC on the other hand lacks the support framework of proprietary solutions, but is open source and handles large datasets well. This allows data the size of which users would normally encounter (500–2,000 images) to be processed using the highest settings on an average‐specification (“consumer‐grade”) desktop/workstation. Though, whether there is a significant difference in terms of cost between SfM + MVS software solutions largely depends on the project. Having said that, we show that the difference in quantified financial value between software (the higher the better) can differ by a factor close to two (see Table 8). Hence, it is clear that there can be significant differences between software, though in many use cases the difference will be neglectable.

### 4.5 Implications of findings

We argue that confidence in the fine‐grained resolution of drone and SfM + MVS‐based outputs in vegetated areas has been undermined both by lack of ground validation data captured at similar grain size, and diversity in workflows. Indeed, this study builds on the work of Fraser and Congalton (2018) and highlights the need to develop standardized workflows within drone and SfM + MVS‐based research and development. The results detailed herein represent an important step toward enabling the establishment of widespread confidence in the longevity of drone and SfM + MVS‐based workflows for biotic resource management. Standardized workflows should make it possible to attribute and report differences in results between studies to variations in the methodological approach or the system studied and therefore should include factors such as number of replicate image datasets, weather conditions, camera type and settings, flying altitude, and software and settings used. This is necessary as we demonstrate that there are statistically significant differences between replicate image datasets, an effect previously largely overlooked. Centimeter‐level variance in RMSE using replicate image datasets captured within the time span of one hour, under very similar conditions, processed using the *same* workflow limits the confidence of drone‐based SfM + MVS as a simple tool to measure ultra‐fine‐grained changes over time when relying on a single image dataset.

## 5 CONCLUSION

The findings presented in this study have important implications for the application of SfM + MVS in ecology as well as in other fields of Earth and environmental science. We demonstrate that there is a need to rethink the importance of the choice of software, and how SfM + MVS studies are carried out as, up until now, most studies employing an SfM + MVS workflow are not necessarily statistically reproducible. When designing a drone and SfM + MVS‐based study, it is crucial to consider differences between software and how robust the workflow, including software, are by considering the variation in the SfM + MVS‐derived vegetation canopy height measurements between replicate image datasets. To address the latter point, we propose that an SfM + MVS workflow should capture at least one replicate image dataset. This would, at a small cost, improve the reproducibility of the results, which is crucial when monitoring fine‐grained indicators of environmental change over time.

## CONFLICT OF INTEREST

None declared.

## AUTHOR'S CONTRIBUTION

J.F., K.A., C.J.A.M., M.E.W., L.D., and R.B. conceived the ideas and designed the methodology; J.F. and L.D. collected the data; J.F. analyzed the data; J.F. and K.A. led the writing of the manuscript. All authors contributed critically to the drafts and gave final approval for publication.

## Data Availability

Data available from the Dryad Digital Repository: https://doi.org/10.5061/dryad.q7c400k (Forsmoo et al., 2019).

## ENDNOTES

1 State‐of‐the‐art is defined as a software package that has been shown to provide high quality results, which is currently under development.

2 Stability over time. That is, how similar the results are when processing the image dataset multiple times using the same workflow.

## References

[ece35443-bib-0001] 3DFlow (2018). Tutorial – 3DF Zephyr parameters tuning. Retrieved from https://www.3dflow.net/3df-zephyr-parameters-tuning-guide/

[ece35443-bib-0002] Anderson, K. , & Gaston, K. J. (2013). Lightweight unmanned aerial vehicles will revolutionize spatial ecology. Frontiers in Ecology and the Environment, 11(3), 138–146. 10.1890/120150

[ece35443-bib-0004] Azzola, P. , Cardaci, A. , Mirabella Roberti, G. , & Nannei, V. M. (2019). Uav Photogrammetry for Cultural Heritage Preservation Modeling and Mapping Venetian Walls of Bergamo. ISPRS ‐ International Archives of the Photogrammetry, Remote Sensing and Spatial Information Sciences, XLII‐2/W9, 45–50. 10.5194/isprs-archives-xlii-2-w9-45-2019

[ece35443-bib-0005] Barnhart, T. B. , & Crosby, B. T. (2013). Comparing two methods of surface change detection on an evolving thermokarst using high‐temporal‐frequency terrestrial laser scanning, Selawik River, Alaska. Remote Sensing, 5(6), 2813–2837. 10.3390/rs5062813

[ece35443-bib-0006] Bogunovic, H. , Sonka, M. , Kwon, Y. H. , Kemp, P. , Abramoff, M. D. , & Wu, X. (2014). Multi‐surface and multi‐field co‐segmentation of 3‐D retinal optical coherence tomography. IEEE Transactions on Medical Imaging, 33(12), 2242–2253. 10.1109/TMI.2014.2336246 25020067 PMC4326334

[ece35443-bib-0078] Bretar, F. , & Chehata, N. (2007). Digital terrain model on vegetated areas: Joint use of airborne LiDAR data and optical images. International Archives of Photogrammetry, 36(3/W49A), 19–24.

[ece35443-bib-0008] Colomina, I. , & Molina, P. (2014). Unmanned aerial systems for photogrammetry and remote sensing: A review. ISPRS Journal of Photogrammetry and Remote Sensing, 92, 79–97. 10.1016/j.isprsjprs.2014.02.013

[ece35443-bib-0009] Cunliffe, A. M. , Brazier, R. E. , & Anderson, K. (2016). Ultra‐fine grain landscape‐scale quantification of dryland vegetation structure with drone‐acquired structure‐from‐motion photogrammetry. Remote Sensing of Environment, 183, 129–143. 10.1016/j.rse.2016.05.019

[ece35443-bib-0010] Dalponte, M. , Frizzera, L. , & Gianelle, D. (2018). How to map forest structure from aircraft, one tree at a time. Ecology and Evolution, 8(11), 5611–5618. 10.1002/ece3.4089 29938078 PMC6010772

[ece35443-bib-0012] Dandois, J. , Olano, M. , & Ellis, E. (2015). Optimal altitude, overlap, and weather conditions for computer vision UAV estimates of forest structure. Remote Sensing, 7, 13895–13920. 10.3390/rs71013895

[ece35443-bib-0077] Dandois, J. P. , & Ellis, E. C. (2010). Remote sensing of vegetation structure using computer vision. Remote Sensing, 2(4), 1157–1176. 10.3390/rs2041157

[ece35443-bib-0014] Forsmoo, J. , Anderson, K. , Macleod, C. J. A. , Wilkinson, M. E. , & Brazier, R. (2018). Drone‐based Structure‐from‐Motion photogrammetry captures grassland sward height variability. Journal of Applied Ecology, 55(6), 2587–2599. 10.1111/1365-2664.13148

[ece35443-bib-0015] Forsmoo, J. , Anderson, K. , Macleod, C. J. A. , Wilkinson, M. E. , DeBell, L. , & Brazier, R. E. (2019). Data from: Structure from motion photogrammetry in ecology: Does the choice of software matter? Dryad Digital Repository, 10.5061/dryad.q7c400k PMC6912889 31871623

[ece35443-bib-0016] Fourcade, Y. , & Öckinger, E. (2017). Host plant density and patch isolation drive occupancy and abundance at a butterfly's northern range margin. Ecology and Evolution, 7(1), 331–345. 10.1002/ece3.2597 28070296 PMC5216661

[ece35443-bib-0017] Fraser, B. T. , & Congalton, R. G. (2018). Issues in Unmanned Aerial Systems (UAS) data collection of complex forest environments. Remote Sensing, 10(6), 10.3390/rs10060908

[ece35443-bib-0018] Fritch, R. A. , Sheridan, H. , Finn, J. A. , McCormack, S. , & Ó hUallacháin, D. (2017). Enhancing the diversity of breeding invertebrates within field margins of intensively managed grassland: Effects of alternative management practices. Ecology and Evolution, 7(22), 9763–9774. 10.1002/ece3.3302 29188007 PMC5696416

[ece35443-bib-0019] Furukawa, Y. , & Hernández, C. (2015). Multi‐view stereo: A tutorial. Foundations and Trends® in Computer Graphics and Vision, 9(1‐2), 1–148. 10.1561/0600000052

[ece35443-bib-0020] Hoffmann, H. , Nieto, H. , Jensen, R. , Guzinski, R. , Zarco‐Tejada, P. J. , & Friborg, T. (2015). Estimating evapotranspiration with thermal UAV data and two source energy balance models. Hydrology and Earth System Sciences Discussions, 12(8), 7469–7502. 10.5194/hessd-12-7469-2015

[ece35443-bib-0021] Hugenholtz, C. H. , Whitehead, K. , Brown, O. W. , Barchyn, T. E. , Moorman, B. J. , LeClair, A. , … Hamilton, T. (2013). Geomorphological mapping with a small unmanned aircraft system (sUAS): Feature detection and accuracy assessment of a photogrammetrically‐derived digital terrain model. Geomorphology, 194, 16–24. 10.1016/j.geomorph.2013.03.023

[ece35443-bib-0022] Ibáñez, I. , Gornish, E. S. , Buckley, L. , Debinski, D. M. , Hellmann, J. , Helmuth, B. , … Uriarte, M. (2013). Moving forward in global‐change ecology: Capitalizing on natural variability. Ecology and Evolution, 3(1), 170–181. 10.1002/ece3.433 PMC3568852 23404535

[ece35443-bib-0023] James, M. R. , & Robson, S. (2014). Mitigating systematic error in topographic models derived from UAV and ground‐based image networks. Earth Surface Processes and Landforms, 39, 1413–1420. 10.1002/esp.3609

[ece35443-bib-0024] James, M. R. , Robson, S. , d'Oleire‐Oltmanns, S. , & Niethammer, U. (2017). Optimising UAV topographic surveys processed with structure‐from‐motion: Ground control quality, quantity and bundle adjustment. Geomorphology, 280, 51–66. 10.1016/j.geomorph.2016.11.021

[ece35443-bib-0025] James, M. R. , Robson, S. , & Smith, M. W. (2017). 3‐D uncertainty‐based topographic change detection with structure‐from‐motion photogrammetry: Precision maps for ground control and directly georeferenced surveys. Earth Surface Processes and Landforms, 42(12), 1769–1788. 10.1002/esp.4125

[ece35443-bib-0026] Javernick, L. , Brasington, J. , & Caruso, B. (2014). Modeling the topography of shallow braided rivers using Structure‐from‐Motion photogrammetry. Geomorphology, 213, 166–182. 10.1016/j.geomorph.2014.01.006

[ece35443-bib-0027] Lague, D. , Brodu, N. , & Leroux, J. (2013). Accurate 3D comparison of complex topography with terrestrial laser scanner: Application to the Rangitikei canyon (N‐Z). ISPRS Journal of Photogrammetry and Remote Sensing, 82, 10–26. 10.1016/j.isprsjprs.2013.04.009

[ece35443-bib-0029] Lesak, A. A. , Radeloff, V. C. , Hawbaker, T. J. , Pidgeon, A. M. , Gobakken, T. , & Contrucci, K. (2011). Modeling forest songbird species richness using LiDAR‐derived measures of forest structure. Remote Sensing of Environment, 115(11), 2823–2835. 10.1016/j.rse.2011.01.025

[ece35443-bib-0030] Lisein, J. , Pierrot‐Deseilligny, M. , Bonnet, S. , & Lejeune, P. (2013). A photogrammetric workflow for the creation of a forest canopy height model from small unmanned aerial system imagery. Forests, 4, 922–944. 10.3390/f4040922

[ece35443-bib-0031] Liu, J. , Engel, B. A. , Wang, Y. , Wu, Y. , Zhang, Z. , & Zhang, M. (2019). Runoff response to soil moisture and micro‐topographic structure on the plot scale. Scientific Reports, 9(1), 1–13. 10.1038/s41598-019-39409-6 30796348 PMC6385309

[ece35443-bib-0032] Lowe, D. G. (2004). Distinctive image features from scale‐invariant keypoints. International Journal of Computer Vision, 60(2), 91–110.

[ece35443-bib-0033] Lucieer, A. , Robinson, S. , Turner, D. , Harwin, S. , & Kelcey, J. (2012). Using a Micro‐Uav for ultra‐high resolution multi‐sensor observations of Antarctic Moss Beds. ISPRS ‐ International Archives of the Photogrammetry, Remote Sensing and Spatial Information Sciences, XXXIX‐B1, 429–433. 10.5194/isprsarchives-XXXIX-B1-429-2012

[ece35443-bib-0034] Lucieer, A. , Turner, D. , King, D. H. , & Robinson, S. (2014). Using an unmanned aerial vehicle (UAV) to capture micro‐topography of antarctic moss beds. International Journal of Applied Earth Observation and Geoinformation, 27, 53–62. 10.1016/j.jag.2013.05.011

[ece35443-bib-0035] Luoto, M. , Toivonen, T. , & Heikkinen, R. K. (2002). Prediction of total and rare plant species richness in agricultural landscapes from satellite images and topographic data. Landscape Ecology, 17, 195–217.

[ece35443-bib-0036] Luscombe, D. J. , Anderson, K. , Gatis, N. , Wetherelt, A. , Grand‐Clement, E. , & Brazier, R. E. (2015). What does airborne LiDAR really measure in upland ecosystems? Ecohydrology, 8(4), 584–594. 10.1002/eco.1527

[ece35443-bib-0037] Magtalas, M. S. L. Y. , Aves, J. C. L. , & Blanco, A. C. (2016). Georeferencing UAS derivatives through point cloud registration with archived lidar datasets. ISPRS Annals of Photogrammetry, Remote Sensing and Spatial Information Sciences, IV, 20–21. 10.5194/isprs-annals-IV-2-W1-195-2016

[ece35443-bib-0038] Mancini, F. , Dubbini, M. , Gattelli, M. , Stecchi, F. , Fabbri, S. , & Gabbianelli, G. (2013). Using unmanned aerial vehicles (UAV) for high‐resolution reconstruction of topography: The structure from motion approach on coastal environments. Remote Sensing, 5(12), 6880–6898. 10.3390/rs5126880

[ece35443-bib-0039] Matlab (2017). boxplot. [online]. Retrieved from https://uk.mathworks.com/help/stats/boxplot.html

[ece35443-bib-0040] McCauley, L. A. , Ribic, C. A. , Pomara, L. Y. , & Zuckerberg, B. (2017). The future demographic niche of a declining grassland bird fails to shift poleward in response to climate change. Landscape Ecology, 32(4), 807–821. 10.1007/s10980-017-0487-x

[ece35443-bib-0041] Mesas‐Carrascosa, F. J. , Clavero Rumbao, I. , Torres‐Sánchez, J. , García‐Ferrer, A. , Peña, J. M. , & López, G. F. (2016). An analysis of the influence of flight parameters in the generation of Unmanned Aerial Vehicle (UAV) Orthomosaicks to Survey Archaeological Areas. Sensors, 16(11), 1838 10.3390/s16111838 PMC5134497 27809293

[ece35443-bib-0043] Mori, A. S. , Tatsumi, S. , & Gustafsson, L. (2017). Landscape properties affect biodiversity response to retention approaches in forestry. Journal of Applied Ecology, 54(6), 1627–1637. 10.1111/1365-2664.12888

[ece35443-bib-0044] Mügler, C. , Planchon, O. , Patin, J. , Weill, S. , Silvera, N. , Richard, P. , & Mouche, E. (2011). Comparison of roughness models to simulate overland flow and tracer transport experiments under simulated rainfall at plot scale. Journal of Hydrology, 402(1–2), 25–40. 10.1016/j.jhydrol.2011.02.032

[ece35443-bib-0045] Müller, J. , Brandl, R. , Brändle, M. , Förster, B. , de Araujo, B. C. , Gossner, M. M. , … Seibold, S. (2018). LiDAR‐derived canopy structure supports the more‐individuals hypothesis for arthropod diversity in temperate forests. Oikos, 127(6), 814–824. 10.1111/oik.04972

[ece35443-bib-0046] Obanawa, H. , & Hayakawa, Y. S. (2015). High‐resolutional topographic survey using small UAV and SfM‐MVS technologies in hardly accessible area. The international symposium on cartography in internet and ubiquitous environments, 2015, 17–19th March, Tokyo.

[ece35443-bib-0047] O'Connor, J. , Smith, M. J. , & James, M. R. (2017). Cameras and settings for aerial surveys in the geosciences. Progress in Physical Geography, 41(3), 325–344. 10.1177/0309133317703092

[ece35443-bib-0048] Ouédraogo, M. M. , Degré, A. , Debouche, C. , & Lisein, J. (2014). The evaluation of unmanned aerial system‐based photogrammetry and terrestrial laser scanning to generate DEMs of agricultural watersheds. Geomorphology, 214, 339–355. 10.1016/j.geomorph.2014.02.016

[ece35443-bib-0049] Peel, H. , Luo, S. , Cohn, A. G. , & Fuentes, R. (2018). Localisation of a mobile robot for bridge bearing inspection. Automation in Construction, 94, 244–256. 10.1016/j.autcon.2018.07.003

[ece35443-bib-0050] Phinn, S. R. , Menges, C. , Hill, G. J. E. , & Stanford, M. (2000). Optimizing remotely sensed solutions for monitoring, modeling, and managing coastal environments. Remote Sensing of Environment, 73(2), 117–132. 10.1016/S0034-4257(00)00087-0

[ece35443-bib-0052] Raeva, P. L. , Filipova, S. L. , & Filipov, D. G. (2016). Volume computation of a stockpile – a study case comparing gps and uav measurements in an open pit quarry. ISPRS ‐ International Archives of the Photogrammetry, Remote Sensing and Spatial Information Sciences, XLI, 999–1004. 10.5194/isprsarchives-XLI-B1-999-2016

[ece35443-bib-0053] Remondino, F. , Barazzetti, L. , Nex, F. , Scaioni, M. , & Sarazzi, D. (2011). UAV photogrammetry for mapping and 3D modeling – current status and future perspectives. ISPRS ‐ International Archives of the Photogrammetry, Remote Sensing and Spatial Information Sciences, XXXVIII, 14–16. 10.5194/isprsarchives-XXXVIII-1-C22-25-2011

[ece35443-bib-0054] Remondino, F. , Del Pizzo, S. , Kersten, T. P. , Troisi, S. (2012). Low‐cost and open‐source solutions for automated image orientation – A critical overview In Ioannides M., Fritsch D., Leissner J., Davies R., Remondino F., & Caffo R. (Eds.), Progress in cultural heritage preservation. EuroMed 2012. Lecture Notes in Computer Science (vol. 7616). Berlin, Heidelberg, Germany: Springer.

[ece35443-bib-0055] Remondino, F. , Nocerino, E. , Toschi, I. , & Menna, F. (2017). A critical review of automated photogrammetric processing of large datasets. ISPRS ‐ International Archives of the Photogrammetry, Remote Sensing and Spatial Information Sciences, XLII‐2/W5, 591–599. 10.5194/isprs-archives-XLII-2-W5-591-2017

[ece35443-bib-0056] Ridding, L. E. , Redhead, J. W. , & Pywell, R. F. (2015). Fate of semi‐natural grassland in England between 1960 and 2013: A test of national conservation policy. Global Ecology and Conservation, 4, 516–525. 10.1016/j.gecco.2015.10.004

[ece35443-bib-0058] Rupnik, E. , Daakir, M. , & Pierrot Deseilligny, M. (2017). MicMac – A free, open‐source solution for photogrammetry. Open Geospatial Data, Software and Standards, 2(1), 14 10.1186/s40965-017-0027-2

[ece35443-bib-0059] Ryan, J. C. , Hubbard, A. L. , Box, J. E. , Todd, J. , Christoffersen, P. , Carr, J. R. , … Snooke, N. (2015). UAV photogrammetry and structure from motion to assess calving dynamics at Store Glacier, a large outlet draining the Greenland ice sheet. Cryosphere, 9(1), 1–11. 10.5194/tc-9-1-2015

[ece35443-bib-0060] Smith, M. W. , Carrivick, J. L. , & Quincey, D. J. (2016). Structure from motion photogrammetry in physical geography. Progress in Physical Geography, 40(2), 247–275. 10.1177/0309133315615805

[ece35443-bib-0061] Stewart, K. E. J. , Bourn, N. A. D. , & Thomas, J. A. (2001). An evaluation of three quick methods commonly used to assess sward height in ecology. Journal of Applied Ecology, 38(5), 1148–1154. 10.1046/j.1365-2664.2001.00658.x

[ece35443-bib-0062] Tao, W. , Lei, Y. , & Mooney, P. (2011). Dense point cloud extraction from UAV captured images in forest area In Proc. 2011 IEEE Int. Conf. Spat. Data Min. Geogr. Knowl. Serv (pp. 389–392). 10.1109/ICSDM.2011.5969071

[ece35443-bib-0063] Thompson, S. E. , Katul, G. G. , & Porporato, A. (2010). Role of microtopography in rainfall‐runoff partitioning: An analysis using idealized geometry. Water Resources Research, 46(7), 1–11. 10.1029/2009WR008835

[ece35443-bib-0064] Tonkin, T. N. , Midgley, N. G. , Graham, D. J. , & Labadz, J. C. (2014). The potential of small unmanned aircraft systems and structure‐from‐motion for topographic surveys: A test of emerging integrated approaches at Cwm Idwal, North Wales. Geomorphology, 226, 35–43. 10.1016/j.geomorph.2014.07.021

[ece35443-bib-0065] Tournadre, V. , Pierrot‐Deseilligny, M. , & Faure, P. H. (2014). Uav photogrammetry to monitor dykes – Calibration and comparison to terrestrial lidar. ISPRS ‐ International Archives of the Photogrammetry, Remote Sensing and Spatial Information Sciences, XL‐3/W1, 143–148. 10.5194/isprsarchives-XL-3-W1-143-2014

[ece35443-bib-0066] Tournadre, V. , Pierrot‐Deseilligny, M. , & Faure, P. H. (2015). Uav linear photogrammetry. ISPRS ‐ International Archives of the Photogrammetry, Remote Sensing and Spatial Information Sciences, XL‐3/W3, 327–333. 10.5194/isprsarchives-XL-3-W3-327-2015

[ece35443-bib-0067] Turner, D. , Lucieer, A. , & Watson, C. (2012). An automated technique for generating georectified mosaics from ultra‐high resolution Unmanned Aerial Vehicle (UAV) imagery, based on Structure from Motion (SfM) Point Clouds. Remote Sensing, 4, 1392–1410. 10.3390/rs4051392

[ece35443-bib-0068] Vassena, G. , & Clerici, A. (2018). Open pit mine 3D mapping by tls and digital photogrammetry: 3D model update thanks to a slam based approach. International Archives of the Photogrammetry, Remote Sensing and Spatial Information Sciences, 42(2), 1145–1148. 10.5194/isprs-archives-XLII-2-1145-2018

[ece35443-bib-0069] Verhoeven, G. (2017). BRDF and its impact on aerial archaeological photography. Archaeological Prospection, 24(2), 133–140. 10.1002/arp.1559

[ece35443-bib-0070] Verhoeven, G. , Karel, W. , Štuhec, S. , Doneus, M. , Trinks, I. , & Pfeifer, N. (2015). Mind your grey tones‐Examining the influence of decolourization methods on interest point extraction and matching for architectural image‐based modelling. International Archives of the Photogrammetry, Remote Sensing and Spatial Information Sciences, 40, 307–314. 10.5194/isprsarchives-XL-5-W4-307-2015

[ece35443-bib-0071] Verhoeven, G. , & Vermeulen, F. (2016). Engaging with the canopy‐multi‐dimensional vegetation mark visualisation using archived aerial images. Remote Sensing, 10.3390/rs8090752

[ece35443-bib-0073] Wang, Q. , Wu, L. , Chen, S. , Shu, D. , Xu, Z. , Li, F. , & Wang, R. (2014). Accuracy evaluation of 3D geometry from low‐attitude UAV collections A case at Zijin Mine. ISPRS ‐ International Archives of the Photogrammetry, Remote Sensing and Spatial Information Sciences, XL‐4, 297–300. 10.5194/isprsarchives-XL-4-297-2014

[ece35443-bib-0074] Waring, P. (1992). Slipping a disc in the grass. Butterfly Conservation News, 50, 51–53.

[ece35443-bib-0079] Yang, W. , Ni-Meister, W. , & Lee, S. (2010). Assessment of the impacts of surface topography, off-nadir pointing and vegetation structure on vegetation lidar waveforms using an extended geometric optical and radiative transfer model. Remote Sensing of Environment, 115(11), 2810–2822. 10.1016/j.rse.2010.02.021

[ece35443-bib-0076] Zahawi, R. A. , Dandois, J. P. , Holl, K. D. , Nadwodny, D. , Reid, J. L. , & Ellis, E. C. (2015). Using lightweight unmanned aerial vehicles to monitor tropical forest recovery. Biological Conservation, 186, 287–295. 10.1016/j.biocon.2015.03.031

