# Supplementary material for: Structure from motion photogrammetry in ecology: Does the choice of software matter?
Source: Ecol Evol. 2019 Sep 30;9(23):12964–79. doi: 10.1002/ece3.5443 (PMC6912889; doi:10.1002/ece3.5443)
Supplement: Supplementary file 1 [file ECE3-9-12964-s001.docx]

Structure from motion photogrammetry: does the choice of software matter for Ecology [Appendix]

To understand the robustness of the software better, the significant differences between the resulting dense point clouds for each of the three replicate image datasets were computed using the M3C2 method (Lague et al. 2013). This was carried out for each software (n=4) using Cloudcompare (ver. 2.9.1) (see Figures S1-S2).

Figure S1

Figure S2

# Reproducibility across software

To understand the robustness of SfM-MVS-based workflows better, the significant differences between the resulting dense point clouds were computed using the M3C2 method (Lague et al. 2013). This was carried out between each of the software (n=4) and the second replicate image dataset using Cloudcompare (ver. 2.9.1) (see Table S3-S4).

Figure S3

Figure S4

*KEY STATISTICS*

An overview of three different components which can give the reader a frame of reference against which other datasets can be compared. The point cloud density – or the number of points, is not necessarily a robust indicator of quality. However, it can provide a rough gauge for the quality of processing settings used – and conversely what one can expect following the workflow outlined herein. Image residual (pixels) is the mean local error in image alignment, as estimated by the bundle adjustment (Bogunovic et al. 2014, James et al. 2017; Forsmoo et al. 2018). GCP residuals show the difference between measured coordinates and the corresponding coordinates within the SfM+MVS-derived 3D model (James et al. 2017). As a rough guideline, one tries to aim for an image residual below half a pixel, and a GCP residual below 2 cm, though the requirements differ between use cases.

“Medium” settings

An overview of three different components which can give the reader a frame of reference against which other datasets can be compared. Table S1 allows comparison between software, and in particular encourages the identification of absolute and relative difference between replicate image datasets. This is for the “Medium” quality settings.

Table S1

“Low” settings

An overview of three different components which can give the reader a frame of reference against which other datasets can be compared. Table S2 allows comparison between software, and in particular encourages the identification of absolute and relative difference between replicate image datasets. This is for the “Low” quality settings.

Table S2

REPLICATE IMAGE DATASETS

A boxplot of the RMSE for Pix4D, Photoscan, 3DFlow and MICMAC for each of the three image datasets is shown in Figure S5. The median RMSE of the SfM+MVS derived sward height is consistently reduced when using higher quality settings when compared to sward height validation data (n = 228).

## Figure S5

Sward height models derived from a SfM+MVS workflow were compared to ground validation sward height models, i.e. DSMs (see Figure 9). The SfM+MVS derived models are compared in terms of RMSE and R^2^. The RMSE ranged from 3.7 cm to 6.1 cm for MICMAC and 3DFlow, respectively, seen over the three replicate image datasets. The correlation coefficient (R^2^) was calculated as the correlation between validation sward height and the sward height measured using the proposed SfM+MVS workflow. Using a paired t-test it was found that there was a statistically significant difference between the model with lowest RMSE and the model with the highest RMSE for the first, second and third replicate image datasets, respectively. Whilst improvements are significant in statistical terms, the differences, given the magnitude, are minimally important in practice. The replicate image datasets are in order – 1 to 3, from left to right (see Figure S6).

Figure S6

Sward height models derived from a SfM+MVS workflow were compared to ground validation sward height models, i.e. DSMs (see Figure 10). The SfM+MVS derived models are compared in terms of RMSE and R^2^. The RMSE ranged from 3.9 cm to 76.2 cm for 3DFlow and Pix4D, respectively, seen over the three replicate image datasets. The correlation coefficient (R^2^) was calculated as the correlation between validation sward height and the sward height measured using the proposed SfM+MVS workflow. Using a paired t-test it was found that there was a statistically significant difference between the model with lowest RMSE and the model with the highest RMSE for the first, second and third replicate image datasets, respectively. The replicate image datasets are in order – 1 to 3, from left to right (see Figure S7).

Figure S7

Table S1 Overview of three variables of interest, i) point cloud # points, ii) image residual and iii) GCP residual for each software (n=4) and replicate image dataset (n=3) using “Medium” quality settings

Table S2 Overview of three variables of interest, i) point cloud # points, ii) image residual and iii) GCP residual for each software (n=4) and replicate image dataset (n=3) using “Low” quality settings

Figure S1 Spatial distribution of significant changes between replicate image datasets (n=3) for four software (Photoscan, 3DFlow, Pix4D, MICMAC) at “Medium” quality settings, respectively.

Figure S2 Spatial distribution of significant changes between replicate image datasets (n=3) for four software (Photoscan, 3DFlow, Pix4D, MICMAC) at “Low” quality settings, respectively.

**Figure S3** Spatial distribution of significant changes between software (n=4) for one replicate image dataset (#2) and “Medium” quality settings, respectively.

**Figure S4** Spatial distribution of significant changes between software (n=4) for one replicate image dataset (#2) and “Low” quality settings, respectively.

## Figure S5 Boxplot of the RMSE of the SfM+MVS derived sward heights generated using the three replicate image datasets, compared to sward height validation data. The data on the x-axis are labelled according to replicate image dataset (1-3), and validation data (sward height). (
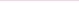
) indicates the median (RMSE), (
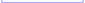
, lower and upper) represents the 25th and 75th percentiles, respectively, (
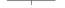
) shows the minimum and maximum data point value (MATLAB (a), 2017).

Figure S6 “Medium” settings. The Root Mean Square Error (m, RMSE) (bar) and R2 (axis reversed) (dot) for each of the SfM+MVS derived DSMs, for each of the three replicate image datasets. The black line indicates the mean RMSE for each of the SfM+MVS software, respectively. The replicate image datasets are in order – 1 to 3, from left to right, for each of the SfM+MVS software tested.

Figure S7 “Low” settings. The Root Mean Square Error (m, RMSE) (bar) and R2 (axis reversed) (dot) for each of the SfM+MVS derived DSMs, for each of the three replicate image datasets. The black line indicates the mean RMSE for each of the SfM+MVS software, respectively. The replicate image datasets are in order – 1 to 3, from left to right, for each of the SfM+MVS software tested.
