# Supplementary figures and images for: Structure from motion photogrammetry in ecology: Does the choice of software matter?
Source: Ecol Evol. 2019 Sep 30;9(23):12964–79. doi: 10.1002/ece3.5443 (PMC6912889; doi:10.1002/ece3.5443)

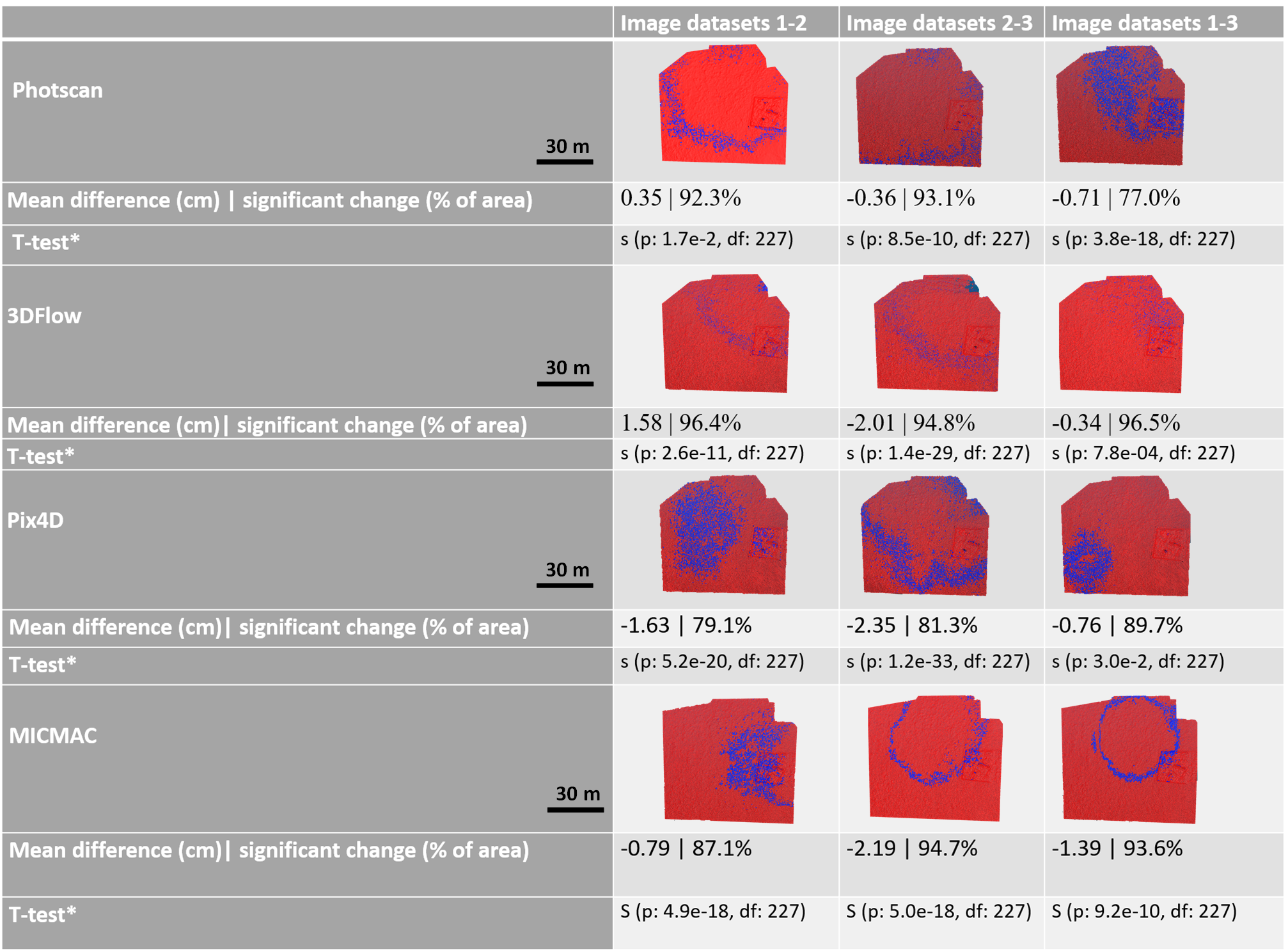

Supplement: Supplementary file 2 [file ECE3-9-12964-s002.tif]

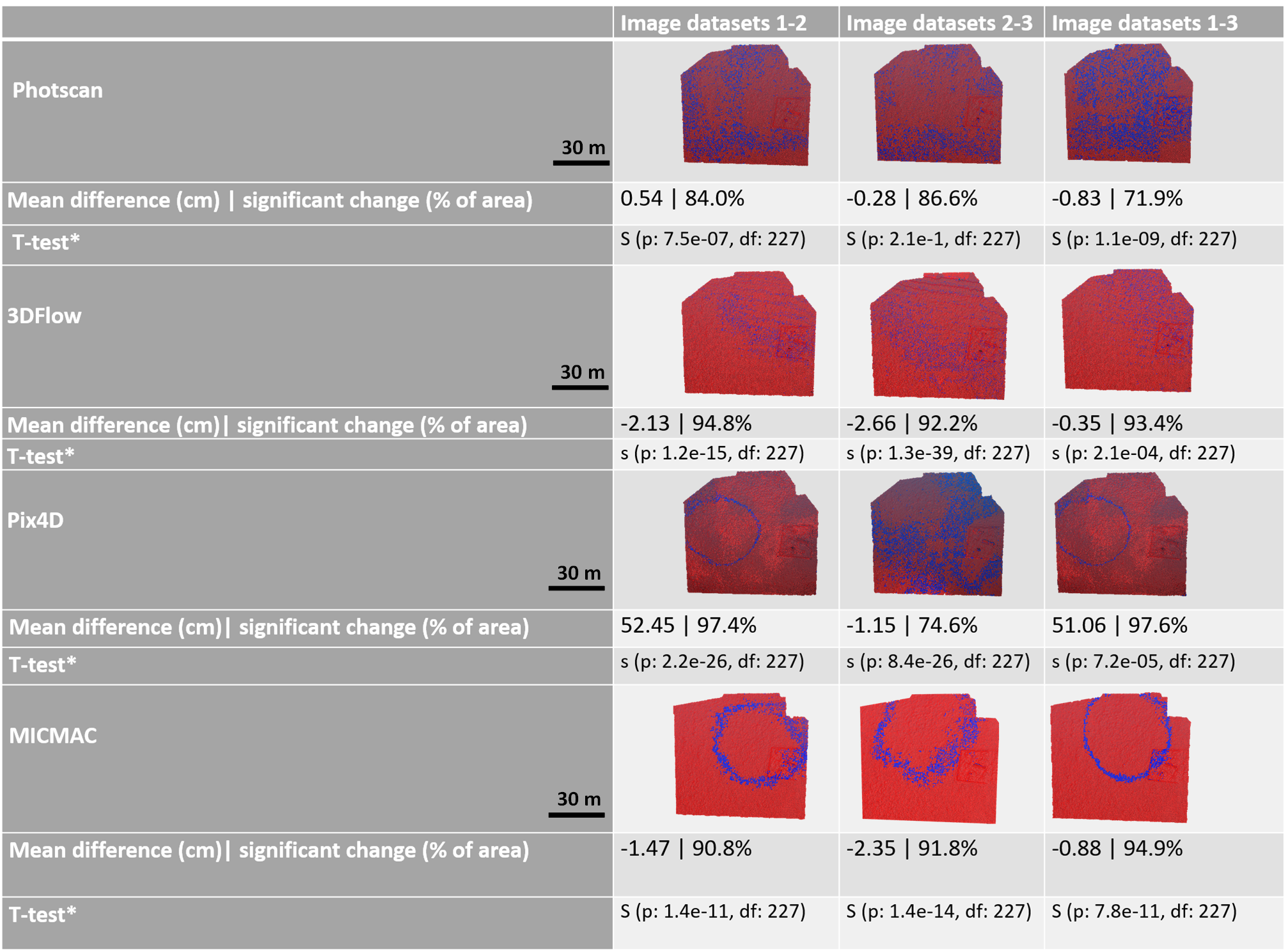

Supplement: Supplementary file 3 [file ECE3-9-12964-s003.tif]

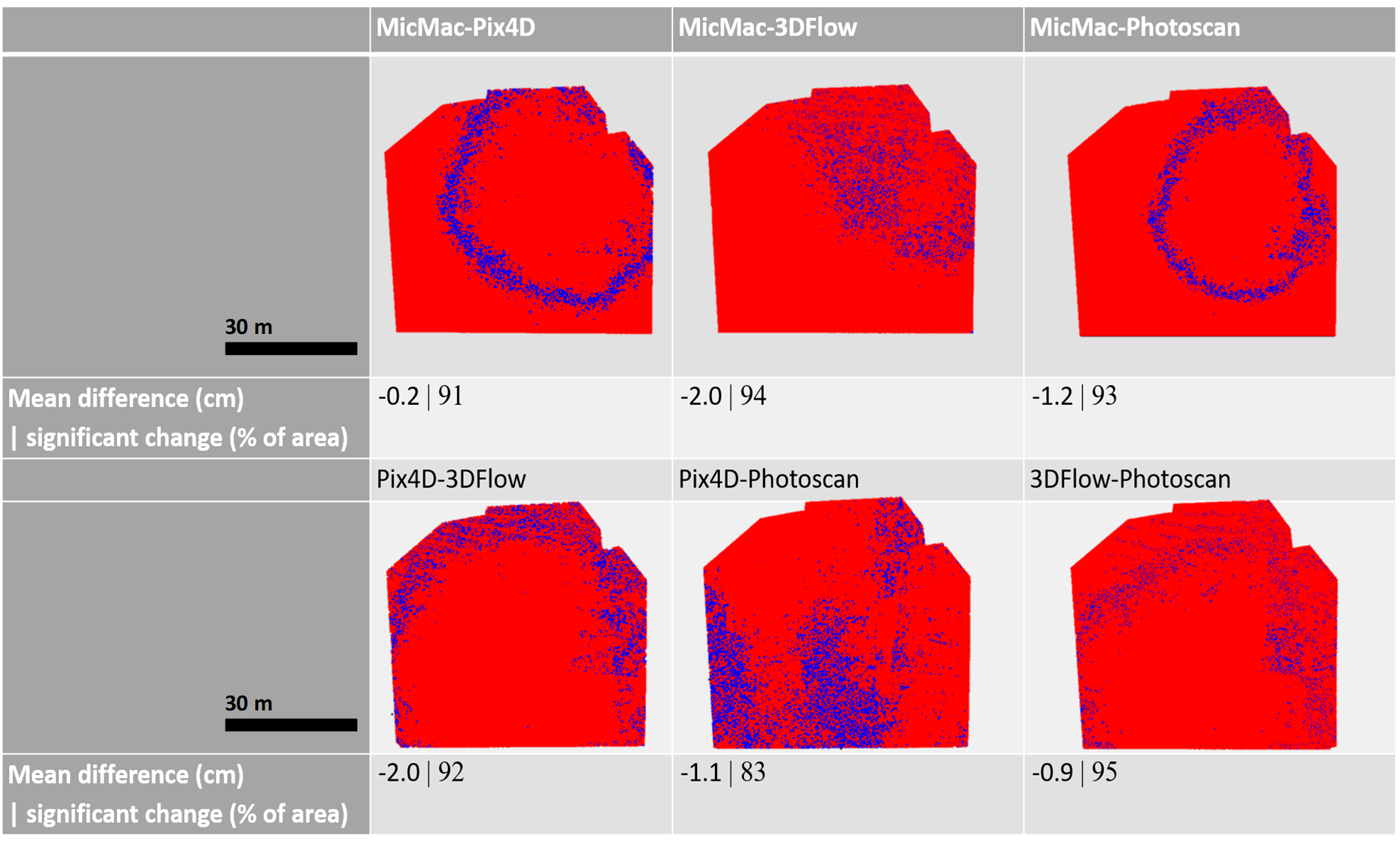

Supplement: Supplementary file 4 [file ECE3-9-12964-s004.tif]

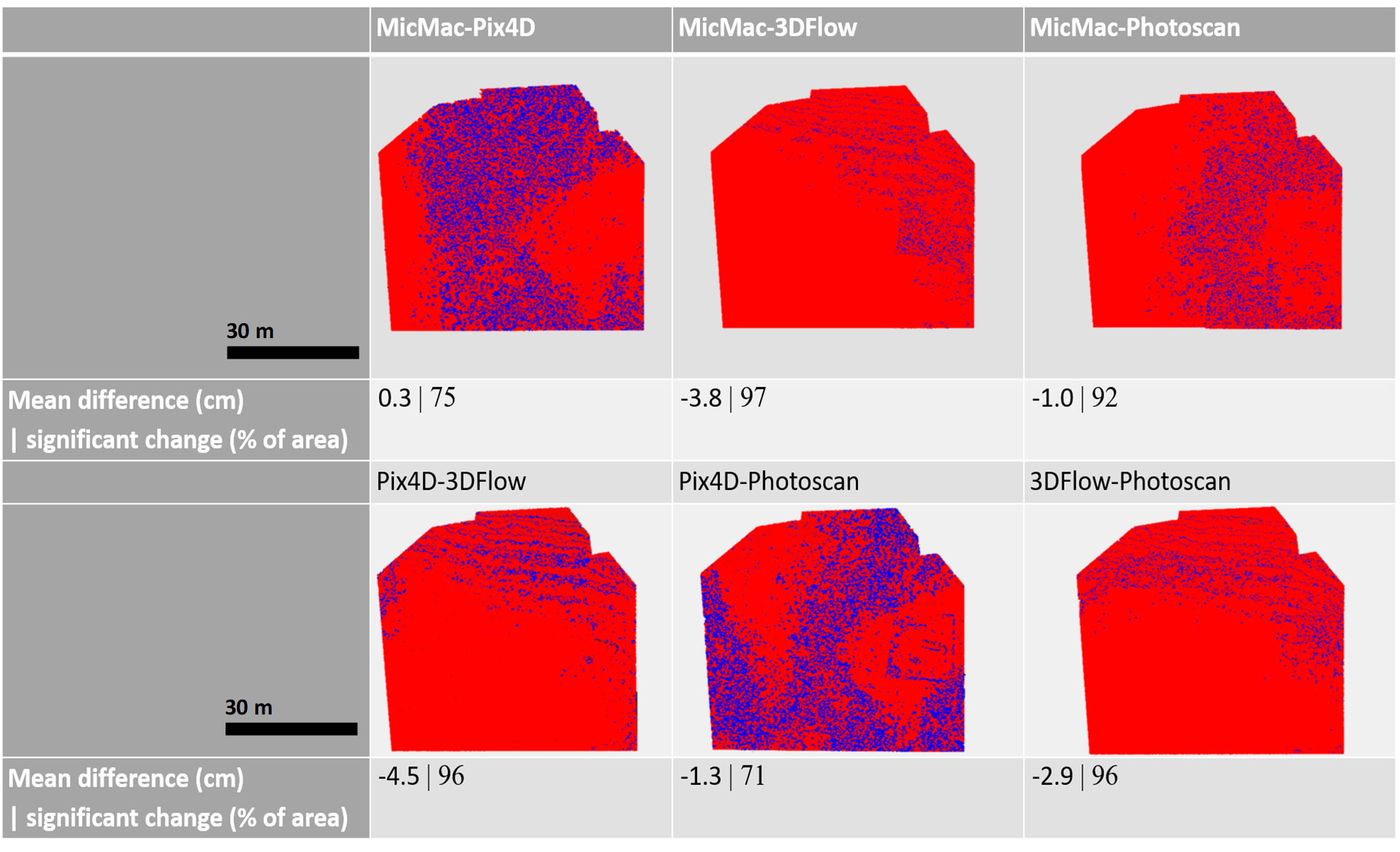

Supplement: Supplementary file 5 [file ECE3-9-12964-s005.tif]

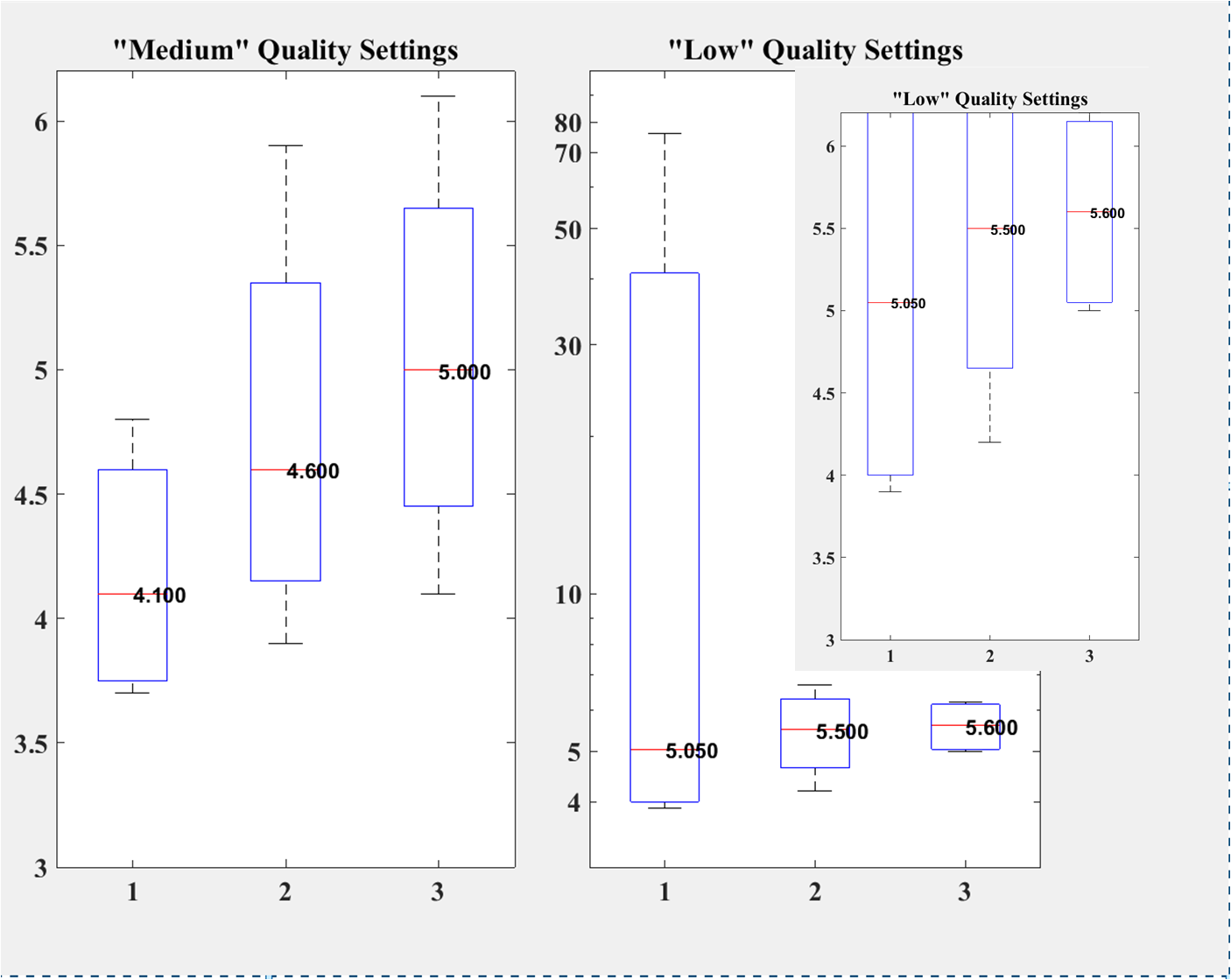

Supplement: Supplementary file 6 [file ECE3-9-12964-s006.tif]

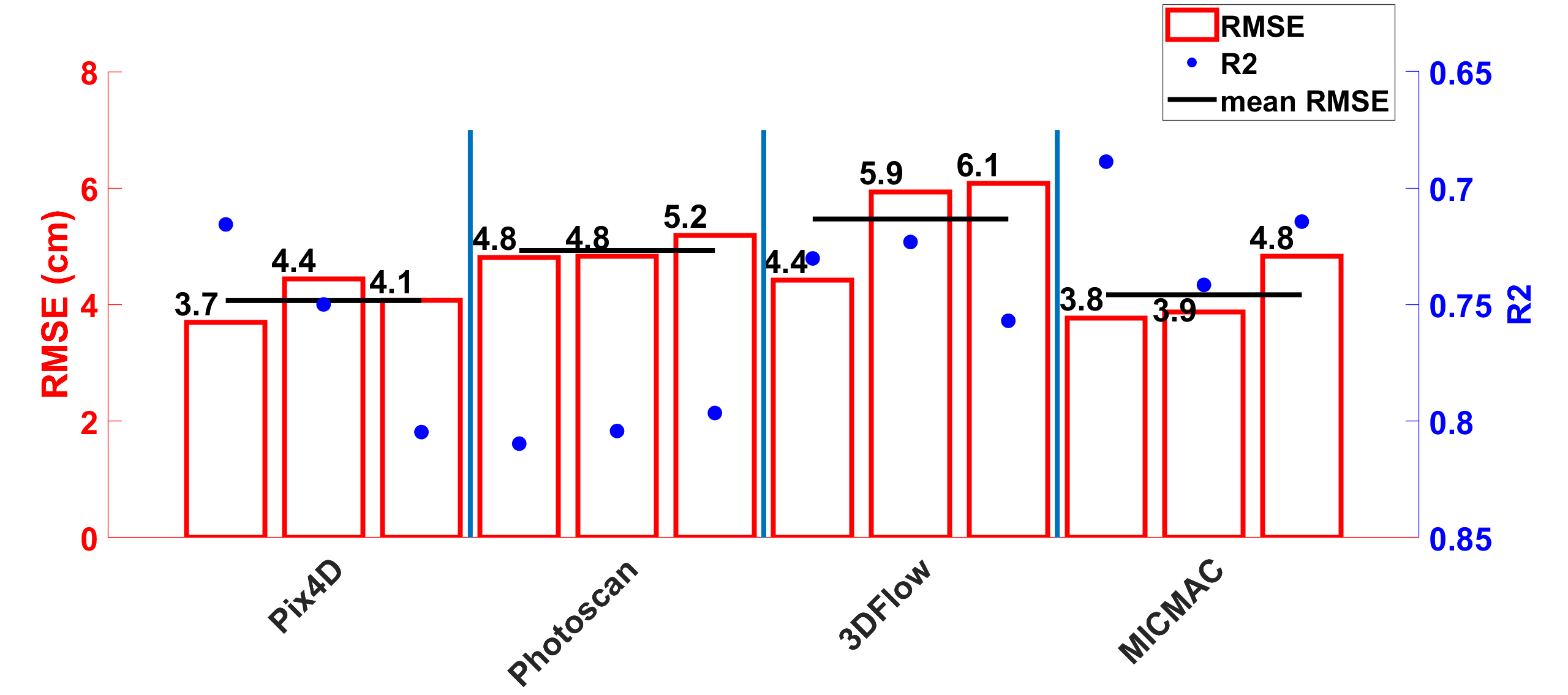

Supplement: Supplementary file 7 [file ECE3-9-12964-s007.tif]

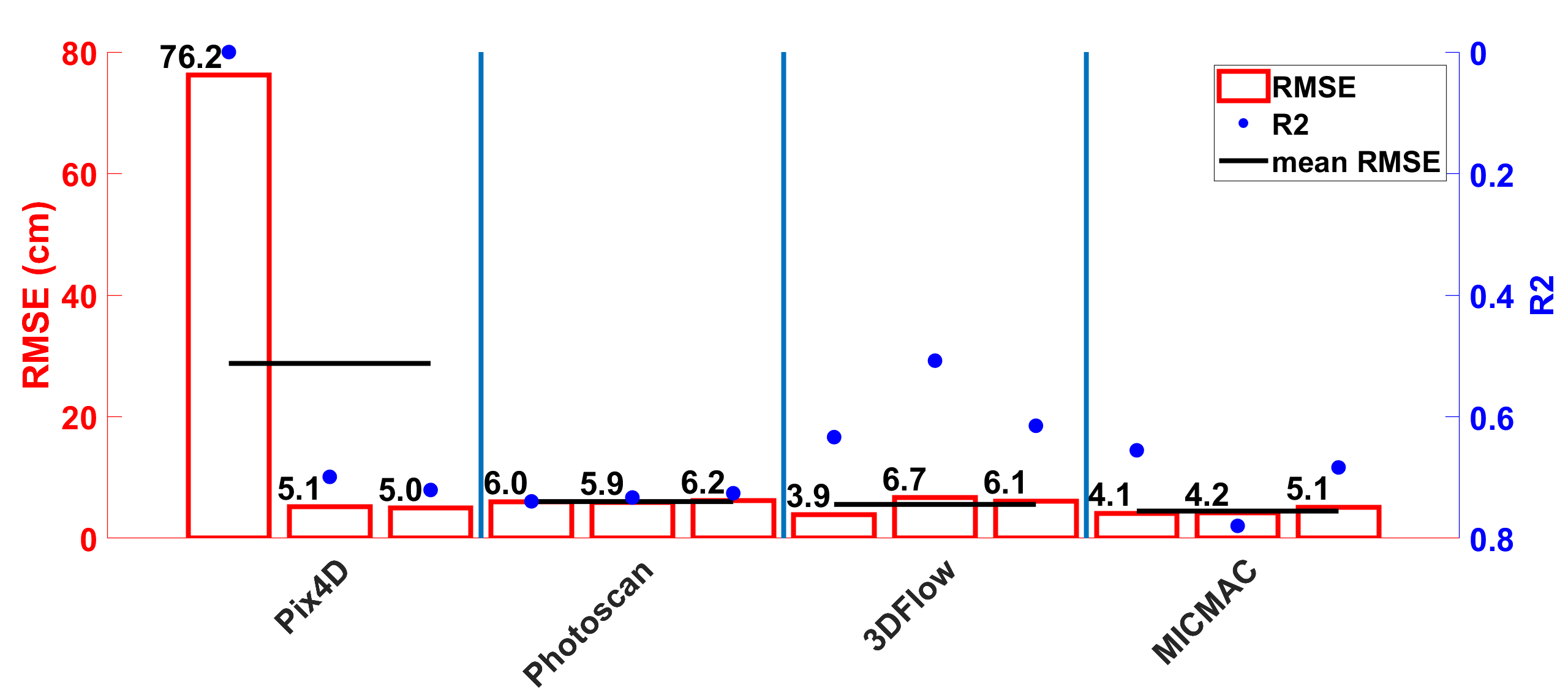

Supplement: Supplementary file 8 [file ECE3-9-12964-s008.tif]
